# Dietary patterns and cardiometabolic health: Clinical evidence and mechanism

**DOI:** 10.1002/mco2.212

**Published:** 2023-02-05

**Authors:** Wenting Wang, Yanfei Liu, Yiwen Li, Binyu Luo, Zhixiu Lin, Keji Chen, Yue Liu

**Affiliations:** ^1^ National Clinical Research Centre for Chinese Medicine Cardiology Xiyuan Hospital China Academy of Chinese Medical Sciences Beijing China; ^2^ Faculty of Medicine The Chinese University of Hong Kong Hong Kong

**Keywords:** cardiometabolic disease, clinical evidence, dietary patterns, gut microbiota, mechanism

## Abstract

For centuries, the search for nutritional interventions to underpin cardiovascular treatment and prevention guidelines has contributed to the rapid development of the field of dietary patterns and cardiometabolic disease (CMD). Numerous studies have demonstrated that healthy dietary patterns with emphasis on food‐based recommendations are the gold standard for extending lifespan and reducing the risks of CMD and mortality. Healthy dietary patterns include various permutations of energy restriction, macronutrients, and food intake patterns such as calorie restriction, intermittent fasting, Mediterranean diet, plant‐based diets, etc. Early implementation of healthy dietary patterns in patients with CMD is encouraged, but an understanding of the mechanisms by which these patterns trigger cardiometabolic benefits remains incomplete. Hence, this review examined several dietary patterns that may improve cardiometabolic health, including restrictive dietary patterns, regional dietary patterns, and diets based on controlled macronutrients and food groups, summarizing cutting‐edge evidence and potential mechanisms for CMD prevention and treatment. Particularly, considering individual differences in responses to dietary composition and nutritional changes in organ tissue diversity, we highlighted the critical role of individual gut microbiota in the crosstalk between diet and CMD and recommend a more precise and dynamic nutritional strategy for CMD by developing dietary patterns based on individual gut microbiota profiles.

## Graphical abstract

The research process for analyzing nutrition and cardiometabolic health is shown in a bottom–up manner. Phase 1: identification of essential nutrients and healthy foods, such as vegetables and fruits, whole grains, and plant‐based oils (tier 4). Phase 2: establishment and development of different types of dietary patterns, such as vegetarian diets, Mediterranean diets, ketogenic diets, calorie restriction, and intermittent fasting (tier 3). Phase 3: exploration of the molecular mechanisms of dietary interventions, including nutrient response pathways, immune regulation, the role of gut microbiota and metabolites, and circadian rhythms (tier 2). Phase 4: providing personalized dietary strategies to cardiometabolic disease patients based on diet–genetic interactions (tier 1).

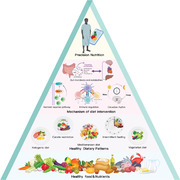

## 1 INTRODUCTION

Cardiometabolic disease (CMD) is a clinical syndrome in which there is a causal relationship between metabolic abnormalities and cardiovascular pathology. There are a range of diseases and conditions classified as CMD, including obesity, type 2 diabetes mellitus (T2DM), and cardiovascular disease (CVD).^1^ According to statistics, 671 million,^2^ 439 million,^3^ and 523 million^4^ people worldwide suffer from obesity, T2DM, and CVD, respectively, resulting in a huge economic burden of over $6.3 trillion.^5^ Behind this phenomenon, a complex interplay of dramatic changes in eating behavior, sub‐optimal nutrition, and atmospheric pollution have contributed to the transformation of CMD from a high‐income country phenomenon to a global health crisis, especially in low‐ and middle‐income countries, with extremely diffuse and devastating effects.^6^, ^7^ There is no doubt that the global challenge of CMD is significant and must be contained before it causes further population health damage and economic loss.

The global health field has fully recognized the priority of the CMD burden, and has developed health policies that target the identification and improvement of cardiometabolic risk factors.^8^ Although these approaches are vital, there is a larger emphasis on prevention strategies that address upstream causes, such as focusing on interventions to influence the determinants of health for all. Evidence shows that the combination of CMD and an unfavorable lifestyle can lead to more than twice the risk of death, while adhering to a healthy lifestyle can offset 63% of the adverse effects of CMD on mortality.^9^ Because everyone needs to eat and drink every day and because nutrition affects almost all physiological processes in the body, dietary interventions are currently the most basic and feasible lifestyle interventions for improving cardiometabolic health and preventing CMD.

**FIGURE 1:**
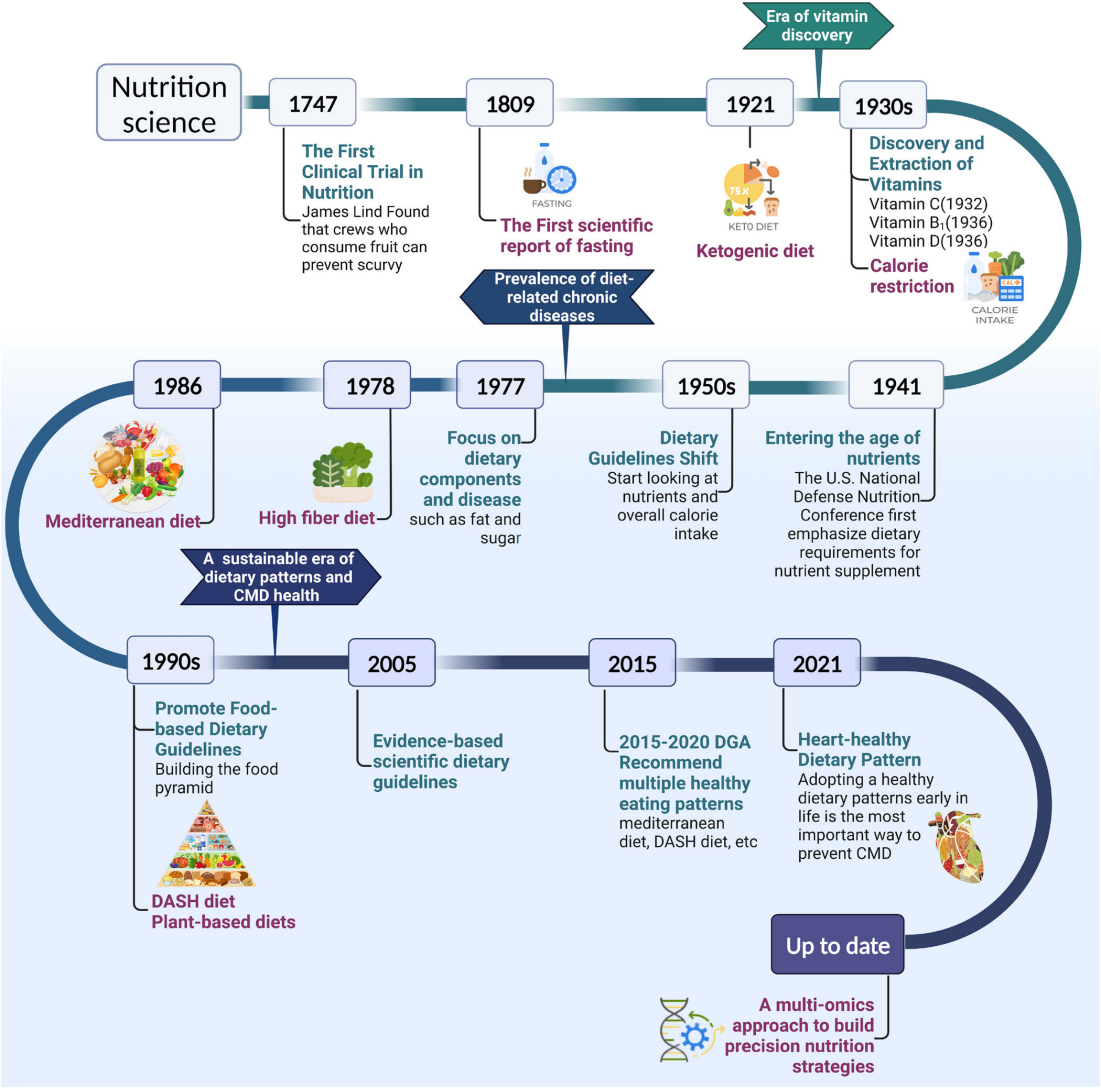
Historical evolution of dietary patterns. From the discovery, isolation, and synthesis of single nutrients to the exploration of the complex biological effects of food and dietary patterns, nutrition science has shifted and advanced in complex thinking. Displayed are the future research priorities of the field. Understanding the evolution of dietary patterns can provide important insights into the current state of diet‐related diseases. Abbreviation: CMD, cardiometabolic diseases; DASH, dietary approaches to stop hypertension

We have only had an understanding of food, nutrition, and disease for a few hundred years, as shown in Figure 1. In 1747, James Lind conducted the world's first controlled experiment in clinical nutrition.^10^ It was about 200 years before the first vitamin, vitamin C, was first isolated and chemically defined.^11^ The next half century witnessed the isolation and synthesis of all the major vitamins and their contribution to the prevention and treatment of nutritional deficiency diseases, resulting in a worldwide popularity of dietary guidelines based on the single‐nutrient theory.^12^ Since the 1970s, the increased burden of chronic non‐communicable diseases (NCDs) has led to a shift in nutrition policy to address chronic diseases. The previously successful reductionist technique for nutrient deficiency illnesses naturally extended, for example, isolated focus on the relationship between total fat, saturated fat, sugar, and coronary artery disease (CAD).^13^, ^14^, ^15^ But this time, nutrients that are so effective in treating nutritional deficiency diseases have not been able to replicate their previous success, a good example being the failure of the “low‐fat diet–heart hypothesis.”^16^, ^17^ People are beginning to realize that the key to diet and disease is not simply explained by nutrition‐focused indicators; in other words, the synergistic effects of different foods and the overall effects of nutrition (i.e., in the form of dietary patterns) are more valuable in addressing the burden of NCDs as it reflects daily dietary behaviors and patterns.

Dietary patterns are defined as the quantities, proportions, variety, or combination of different foods, drinks, and nutrients (when available) in diets, and the frequency with which they are habitually consumed.^18^ Over the past two decades, many different sources and scientifically supported empirical or commercial dietary patterns have been widespread and have inspired a great deal of scientific research related to CMD,^19^, ^20^, ^21^ such as the Mediterranean diet, vegetarian diet, dietary approaches to stop hypertension (DASH) diet, ketogenic diet (KD), etc.^22^, ^23^, ^24^ Current evidence suggests that healthy dietary patterns are the most promising interventions for improving symptoms and reducing the risk of CMD.

An increasing number of clinical studies have suggested that the gut microbiota and microbial metabolites are significantly different between patients with CMD and normal subjects.^25^, ^26^, ^27^, ^28^ A recent study revealed microbiome and metabolome features of the CMD spectrum. Patients with CMD already exhibit microbiota changes such as reduced bacterial cell counts and loss of microbial function in the early stages of their metabolic disorders. These changes continue to drive the development of cardiac lesions.^29^ The composition of gut microbiota is closely related to substrate availability and the intestinal environment, both of which are influenced by diet.^30^, ^31^ Therefore, further enriching clinical and modeling studies and gaining a deeper understanding of the relationship between dietary patterns, gut microbiota, and CMD will help refine nutritional science at the molecular, biological, and metabolite levels.

**FIGURE 2:**
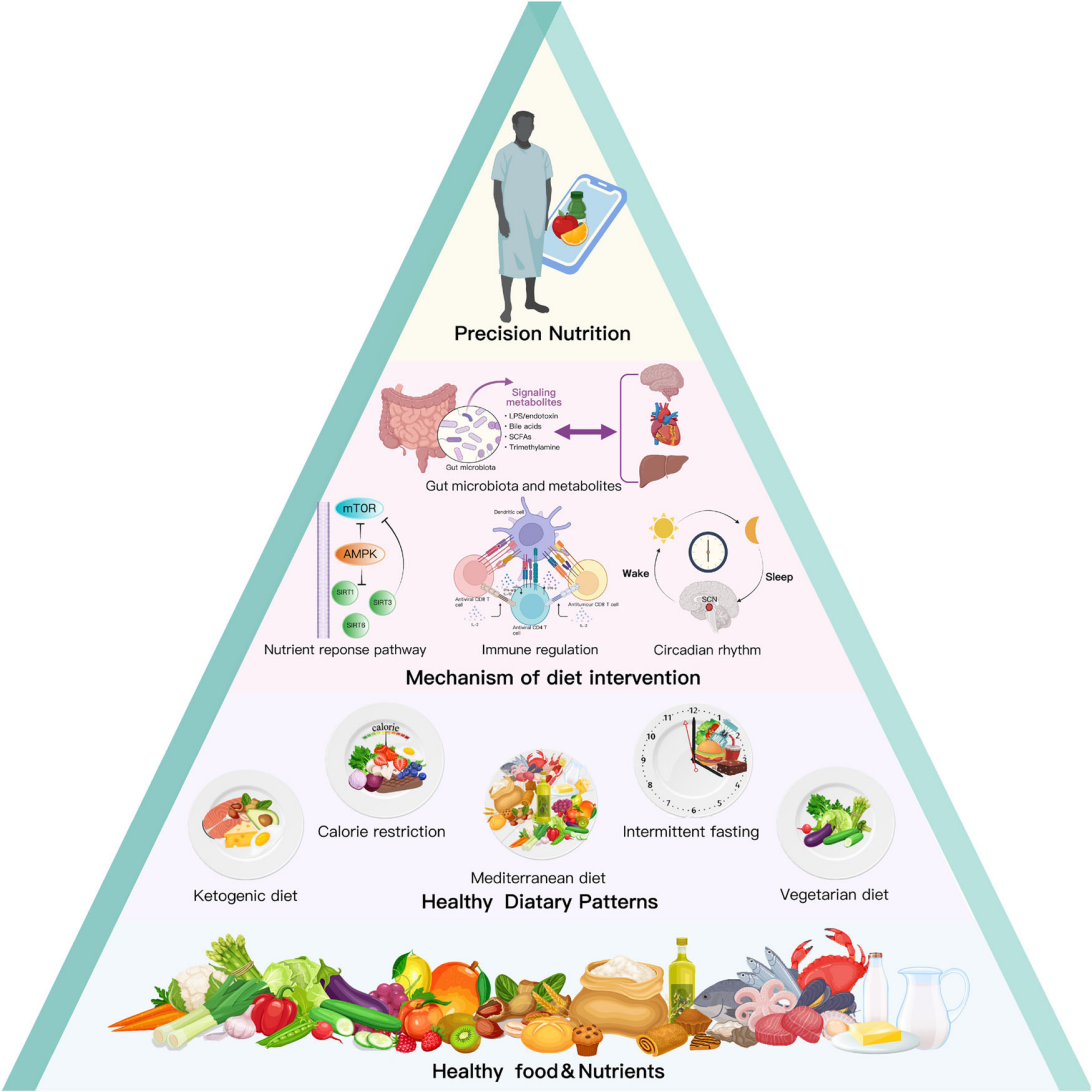
From healthy foods, nutrients, and dietary patterns to cardiometabolic health. The research process for analyzing nutrition and cardiometabolic health is shown in a bottom–up manner. Phase 1: identification of essential nutrients and healthy foods, such as vegetables and fruits, whole grains, and plant‐based oils (tier 4). Phase 2: establishment and development of different types of dietary patterns, such as vegetarian diets, Mediterranean diets, ketogenic diets, calorie restriction, and intermittent fasting (tier 3). Phase 3: exploration of the molecular mechanisms of dietary interventions, including nutrient response pathways, immune regulation, the role of gut microbiota and metabolites, and circadian rhythms (tier 2). Phase 4: providing personalized dietary strategies to cardiometabolic diseases (CMD) patients based on diet–genetic interactions (tier 1). Abbreviation: AMPK, AMP‐activated protein kinase; LPS, lipopolysaccharide; mTOR, mammalian target of rapamycin; SCFA, short‐chain fatty acids; SIRT, Sirtuin

This review article focuses on the latest clinical and mechanistic evidence for improving CMD through dietary patterns, presenting new perspectives and research directions to understand how dietary patterns drive and orchestrate cardiometabolic pathways, as shown in Figure 2.

## 2 DIETARY PATTERNS WITH POTENTIAL FOR CARDIOMETABOLIC HEALTH

The definition of a healthy diet is constantly evolving, reflecting our growing understanding of the roles of different foods, nutrients, and dietary combinations in health.^32^ For nearly 20 years, numerous and increasing clinical and basic studies have developed a number of dietary patterns that can be defined as “heart healthy diets.”^33^ These dietary patterns differ in composition and focus, but all have varying degrees of ability to maintain multiple risk factors, including weight, blood glucose, blood pressure (BP), and blood lipids, within an ideal range. For example, body mass index (BMI) <25kg/m^2^ and waist circumference (WC) ≤88 cm (women)/WC ≤102 cm (men), fasting plasma glucose (FPG) <100 mg/dl, hemoglobin A1c (HbA_1c_) <5.7%, total cholesterol:high‐density lipoprotein cholesterol (TC:HDL‐c) <3.5:1, BP <120/80 mmHg without taking medication, and no signs of CVD.^34^ Here, we focus on three types of dietary patterns according to previous studies and the composition and focus of the diet,^35^, ^36^, ^37^ (1) dietary restrictions, (2) traditional regional diets, and (3) diets based on the control of macronutrient content or foods.

### 2.1 Dietary restriction

Dietary restriction is the most common therapeutic dietary pattern to achieve therapeutic goals for disease by limiting metabolic unfavorable factors. Two main types of implementation strategies are commonly used: one is restriction of overall dietary calories, such as calorie restriction (CR) and fasting, and the other is restriction of macronutrients in food, including dietary protein restriction (PR), dietary carbohydrate restriction, and dietary fat restriction, as shown in Table 1.

**TABLE 1:** Overview of characteristics of dietary restriction

Dietary intervention	Dietary patterns	Characteristic
Calorie restriction	Traditional calorie restriction^38^	25%–30% reduction in average daily calorie intake without compromising the intake of other essential nutrients
	Intermittent calorie restriction^39^	Alternation of severe calorie restriction and regular calorie intake, including alternative day calorie restriction, time‐restricted calorie restriction, etc.
	Low‐calorie diet^40^	Provides 10% less calories per day than the total metabolic expenditure per person, usually 1000–1200 kcal/day, and maintain a balanced diet structure
	Very low‐calorie diet^41^	Provide <800 kcal or less per day, usually in liquid form and with 70–100 g protein/day
Fasting	Intermittent fasting^42^, ^43^	Alternative day fasting: alternate between “a feast day” and “a fast day” at 24‐h intervals
		Time‐restricted fasting: limit food intake to a certain duration per day
		5:2 diet: fasting (continuous or non‐continuous) on 2 days of the week and eating freely on the other 5 days
		Ramadan fasting: abstention from any food and drink from dawn to sunset during the month of Ramadan, with a large meal after sunset and a light meal before dawn
	Long‐term or prolonged fasting^44^	Fasting for 2–21 days or more
	Fasting‐mimicking diet^45^	Low‐calorie low‐protein diet for 5 consecutive days per month, recommended for 1–6 months per year
Dietary protein restriction	Protein restriction^46^	Reduce dietary protein intake without changing average caloric intake
Dietary carbohydrate restriction	Low‐carbohydrate diet^47^	Carbohydrate <130 g/day or <26% total energy
Dietary fat restriction	Low‐fat diet^48^	<30% kcal/day from total fat (<10% of saturated fat)

#### 2.1.1 Calorie restriction

CR is generally defined as a dietary pattern that reduces average daily calorie intake by 25%–30% without affecting the intake of other essential nutrients.^38^ Numerous studies conducted over the past century, including those analyzing yeast, fruit flies, worms, fishes, rodents, and primates, have demonstrated that CR can extend the organism's lifespan by reducing the basal metabolic rate (BMR), suppressing inflammation and oxidative stress, and improving insulin sensitivity.^49^, ^50^ These mechanisms are also applicable to humans. CR has been shown to improve fat distribution and glucolipid metabolism, inhibit oxidative stress and inflammatory damage, and reverse the harmful effects of CMD, such as obesity, T2DM, and atherosclerosis.^51^, ^52^, ^53^, ^54^, ^55^ Moderate CR along with an improved diet quality has been proposed as a way to reduce the risk of CMD and promote healthy aging, as shown in Table 2.

**TABLE 2:** Effect of calorie restriction (CR) on cardiometabolic risk factors in randomized clinical trials (RCTs)

Disease/target residents	Follow‐up time	Improvements in cardiometabolic health	Ref.
Healthy participants (*n* = 46)	12 months	Body composition: body weight↓ BMI↓ fat mass↓ Glucoregulatory factors: fasting insulin↓ Inflammatory biomarkers: TNF‐α: adiponectin ratio↓	^52^
Healthy participants (*n* = 48)	6 months (CALERIE)	Body composition: body weight↓ Glucoregulatory factors: fasting insulin↓	^56^
Healthy participants (*n* = 48)	1 year (CALERIE)	Body composition: body weight↓ BMI↓ fat mass↓	^57^
Healthy participants (*n* = 48)	1 year (CALERIE)	Body composition: fat mass↓ Plasma lipids: TC↓ LDL‐c↓ TG↓ TC:HDL‐c↓ Glucoregulatory factors: HOMA‐IR↓ Inflammatory biomarkers: hs‐CRP↓	^58^
Healthy participants (*n* = 218)	2 years (CALERIE‐2)	Body composition: body weight↓ BMI↓ body fat↓ fat mass↓ BP: SBP↓ DBP↓ Plasma lipids: TC↓ LDL‐c↓ TG↓ TC:HDL‐c↓ HDL‐c↑ Glucoregulatory factors: FPG↓ HOMA‐IR↓ fasting insulin↓ HOMA‐β↓ insulin sensitivity↑ Inflammatory biomarkers: hs‐CRP↓	^59^
Overweight adults (*n* = 35)	6 months	Body composition: body weight↓ VAT volume↓ SAT volume↓	^51^
Overweight or obese females (*n* = 48)	4 weeks	Oxidative stress: F2‐isoprostane↓	^53^
Overweight or obese older adults (BMI of 30–40 kg/m^2^, age >65 years) (*n* = 148)	12 months (CROSS ROADS)	Body composition: body weight↓ body fat↓ Glucoregulatory factors: FPG↓ Plasma lipids: HDL‐c↑	^60^

Abbreviations: BMI, body mass index; BP, blood pressure; DBP, diastolic blood pressure; FPG, fasting plasma glucose; HDL‐c, high‐density lipoprotein cholesterol; HOMA‐IR, homoeostasis model assessment‐estimated‐insulin resistance; hs‐CRP, high sensitivity C‐reactive protein; LDL‐c, low‐density lipoprotein cholesterol; SAT, subcutaneous fat; SBP, systolic blood pressure; TC, total cholesterol; TG, triglyceride; TNF, tumor necrosis factor; VAT, visceral fat; ↓, decrease in the indicated parameter; ↑, increase in the indicated parameter.

Since the Biosphere 2 study,^61^ the amount of evidence supporting the role of CR as a cardiometabolic protector has increased.^62^, ^63^, ^64^ The first large‐scale randomized clinical trial (RCT) on CR (CALERIE‐1) demonstrated that CR for 6–12 months has significant benefits for reducing conventional cardiometabolic risk factors, including improvements in body composition, the lipid profile, blood sugar, and inflammatory markers.^56^, ^57^, ^58^ The subsequent CALERIE‐2 trial further demonstrated that a 2‐year CR intervention (average energy intake reduced by 11.9%) not only improved abnormal cardiometabolic risk factors, but also maintained the positive effects on cardiometabolic profile during the weight stabilization period after weight loss. Even if these risk factors are within normal baseline values, CR interventions can still achieve improvements, implying that long‐term CR prevents the development of CMD.^59^

In addition to the traditional approach of reducing calorie intake outside of every meal, CR can be combined with other lifestyles or dietary patterns to achieve greater metabolic benefits. For example, data from CALERIE‐1 show that 6 months of CR plus exercise reduces the 10‐year risk of CVD by 30%.^65^ The CROSSROADS trial also reported that an 8‐week CR intervention combined with exercise improved the cardiometabolic profile of obese persons aged 65–70 years, including improvements in body weight, FPG, and HDL‐c.^60^ A sub‐analysis of this study also found that the combination of CR and exercise increased the ratio of adiponectin to leptin, effectively reversing the dysfunction of adipose tissue.^66^ Studies by Tang et al.^67^ and da Silva Soares et al.^68^ also demonstrated the positive effects of CR combined with exercise in improving insulin sensitivity, reducing insulin resistance, and preventing muscle atrophy. The above studies are the best evidence for our slogan “eat less, move more.”

Other studies have demonstrated that CR combined with intermittent fasting (IF), which is called intermittent calorie restriction (ICR), can produce the same or even better cardiometabolic benefits as those from continuous calorie restriction (CCR).^39^ This may be related to the fact that it is easier for individuals to commit to ICR than to CCR. In an 8‐week RCT of 88 overweight and obese adults with hypertriglyceridemia, 3 days per week of ICR resulted in larger triglyceride (TG) reductions and appeared to be more successful in reducing insulin resistance than the CCR group.^69^ Additionally, low‐calorie diets (10% less calories per day) developed based on CR and very low‐calorie diets (800 kcal or less per day) have also demonstrated impressive results in the treatment of CMD,^70^ improving body composition, controlling cardiovascular risk factors, and producing positive effects on blood sugar levels.^40^, ^41^, ^71^, ^72^, ^73^

CR without malnutrition is the most effective non‐pharmacological intervention for extending healthy lifespan, slowing aging, and combating CMD. However, in practice, several dietary patterns, including fasting, the Mediterranean diet, and the DASH diet all achieve varying degrees of CR alongside the intervention, and CR can work in synergy with other dietary patterns to optimize metabolism. Most human trials of CR have been conducted primarily in overweight or obese populations with a focus on weight loss rather than age‐related diseases. Its significant advantages in improving body composition make it more suitable for the prevention of CMD risk in overweight or obese patients. Also, the therapeutic effect of CR in patients with T2DM, CVD, and non‐alcoholic fatty liver disease (NAFLD) is largely based on good control of body weight.^74^ Further research is needed to clarify the metabolic benefits of CR in the prevention and treatment of CMD beyond weight loss.

#### 2.1.2 Fasting

Fasting is the intentional cessation of solid meals and stimulants (caffeine, nicotine) for a limited period of time.^44^ In fact, if we strictly adhere to the theory of three meals, we are experiencing 8–10 h of fasting every day. Compared with CR, which must strictly control the types of food intake and monitor energy intake, fasting can be achieved by simply ensuring that no food intake is consumed for a period (>12 h). This simplicity and ease of compliance has contributed to the rapid popularity of fasting as an alternative dietary strategy to CR. The main fasting therapies currently available include IF, long‐term or prolonged fasting (LF), and fasting‐mimicking diets (FMD).

##### Intermittent fasting

Starvation for less than 2 days and meeting alternating fasting and ad libitum food intake.^75^ The most widely studied IF are alternative day fasting (ADF) and time‐restricted fasting/eating (TRF/TRE).^76^ ADF is a fasting pattern in which fasting days and feeding days at 24‐h intervals are alternated.^42^ This alternating fasting behavior causes widespread systemic effects, resulting in changes in metabolic pathways, cellular processes, and hormone secretion,^77^ such as lower blood glucose and higher circulating ketones, as well as increased secretion of glucagon and growth hormone, and ultimately causes a reduction in body weight, visceral fat, lipid levels, and improvements in circulating inflammation and oxidative stress.^78^ Numerous preclinical studies have exhibited that ADF positively impacts obesity,^79^ T2DM,^80^ CVD,^81^ cancer,^82^ and many other chronic diseases. ADF has demonstrated the best ability to extend life compared with other IF regimens.^75^ Two clinical trials observed the metabolic benefits gained from implementing ADF in healthy individuals.^83^, ^84^ ADF has consistently demonstrated significant improvements in body weight, the fat/muscle ratio, glycolipid metabolism, and BP compared with control diets, especially in terms of outstanding weight loss. It also exhibits significant effects on the most lipotoxic androgenic regions that influence the development of CVD,^85^ making it an effective tool for promoting cardiovascular health in patients with CMD. In view of the already promising effects of ADF on weight loss in the general population, more studies have focused on its effects on overweight, obesity, and metabolic abnormalities. The results demonstrated that ADF showed consistent reductions in body weight, body fat mass, and BMI in obese patients, T2DM patients, and those at high CVD risk, and was superior in reducing TC, low‐density lipoprotein cholesterol (LDL‐c), FPG, homoeostasis model assessment‐estimated‐insulin resistance (HOMA‐IR) and high sensitivity C‐reactive protein (hs‐CRP).^86^, ^87^, ^88^, ^89^, ^90^, ^91^ Together with animal studies, these results demonstrate the benefits of ADF in maintaining cardiometabolic health.

TRF/TRE is a dietary pattern that restricts daily food intake to a specific period.^92^ TRF/TRE improves metabolic rhythms and protects the body from metabolic diseases, such as obesity and inflammation, independent of CR.^93^ Almost all current evidence now supports the cardiometabolic protective effect of short‐term TRE, including regulation of body weight, glucolipid metabolism, inflammatory and adipokine secretion, circadian gene expression, and gut microbiota composition, which can be used as a dietary intervention to prevent and treat CMDs such as obesity and T2DM.^94^, ^95^, ^96^, ^97^, ^98^, ^99^, ^100^, ^101^, ^102^, ^103^, ^104^, ^105^, ^106^, ^129^ Recently, Liu et al.^107^ reported that 12 months of TRE combined with CR resulted in better weight loss than CR alone and significantly improved several cardiometabolic parameters, such as fat mass, fasting blood glucose, and lipid levels in patients with obesity. Increasing the length of the TRE intervention and tightly controlling calories may be the best option for patients with obesity.

Furthermore, as a temporal nutritional strategy, many of the health benefits of TRE/TRF arise from the close alignment of the timing of eating with typical metabolite and hormone profiles over the span of 24 h. Therefore, many clinical studies have been designed to determine when eating is most beneficial to health.^94^ For example, Jamshed et al.^108^ showed that 14 weeks of early TRF (eTRF) significantly improved weight loss, BP, the emotional state, and energy in patients with obesity, hinting that early implementation may be the key to achieving more efficient weight loss with TRF. Xie et al.^109^ observed that eTRF (eating between 06:00 and 15:00) had positive effects in lowering the fasting blood glucose level and insulin resistance, reducing body weight and fat mass, and improving inflammation and increasing gut microbial diversity in healthy individuals. In contrast, middle TRF (mTRF, eating between 11:00 and 20:00) did not have these effects. These results are consistent with previous studies demonstrating that eTRF, which is consistent with hormonal rhythms, has a better ameliorating effect on metabolism.^110^, ^111^, ^112^, ^113^, ^114^, ^115^ The effectiveness of the TRF/TRE regimen is largely dependent on its synchronization with daily circadian rhythms.

In addition, the 5:2 diet and Ramadan diet have also demonstrated effectiveness in IF programs for preventing and treating CMD.^116^, ^117^ The 5:2 diet, also listed as periodic fasting, is characterized by two fasting days (consecutive or nonconsecutive) and ad libidum intake for another 5 days.^42^ Several RCTs have shown that the 5:2 diet is more effective in controlling blood glucose in patients with obesity, T2DM, and metabolic syndrome (MetS), and also significantly improves weight, BP, and adiposity factors.^118^, ^119^, ^120^, ^121^, ^122^, ^123^, ^124^, ^125^ Ramadan fasting is one of the five pillars of Islam. During Ramadan, Muslims keep fasting from sunrise to sunset, eating a large meal after sunset and a light meal before dawn.^43^ Studies have shown that Ramadan fasting is significantly related to reduced risks of CMD indicators.^126^, ^127^, ^128^ However, this beneficial effect tends to be short‐lived, and some studies suggest that this fasting regimen may cause an increase in LDL‐c and insulin resistance.^126^ The relatively flexible implementation protocols of the 5:2 diet and the Ramadan diet compared to the ADF and TRF protocols may lead to more variable changes, resulting in greater heterogeneity of study results, as shown in Table 3.

**TABLE 3:** Effect of intermittent fasting (IF) on cardiometabolic risk factors in randomized clinical trials (RCTs)

Dietary intervention	Disease/target population	Follow‐up time	Improvements in cardiometabolic health	Ref.
ADF	Healthy subjects (*n* = 60)	4 weeks	Body composition: body weight↓ BMI↓ fat mass↓ BP: SBP↓ DBP↓	^83^
	Overweight or obese adults (*n* = 31)	8 weeks	Body composition: body weight↓	^86^
	Overweight or obese adults (*n* = 100)	52 weeks	Body composition: body weight↓ Glucoregulatory factors: fasting insulin↓	^87^
	Overweight or obese adults (*n* = 69)	8 weeks	Body composition: body weight↓ BP: SBP↓ DBP↓ Glucoregulatory factors: FPG↓ HOMA‐IR↓ fasting insulin↓	^88^
	Participant with MetS (*n* = 80)	4 months	Body composition: body weight↓ BMI↓ Inflammatory biomarkers: hs‐CRP↓	^89^
	Overweight or obese adults with prediabetes (*n* = 101)	3 months	Body composition: body weight↓ BMI↓ Plasma lipids: TC↓ HDL‐c↓ Glucoregulatory factors: FPG↓	^90^
4 h/6 h TRF	Overweight or obese adults (*n* = 58)	8 weeks	Body composition: body weight↓ Glucoregulatory factors: HOMA‐IR↓ Fasting insulin↓	^96^
8 h TRF	Healthy resistance‐trained males (*n* = 20)	1 year	Body composition: fat mass↓ Plasma lipids: LDL‐c↓ TG↓ HDL‐c↑ Glucoregulatory factors: FGP↓ HOMA‐IR↓ fasting insulin↓ Adipose factor: leptin↓ adiponectin↑ Inflammatory biomarkers: IL‐6↓ IL‐1β↓ TNF‐α↓	^102^
	Overweight or obese adults (*n* = 46)	1 year	Body composition: body weight↓ BP: SBP↓	^105^
	Overweight or obese adults (*n* = 116)	12 weeks	Body composition: body weight↓ ALMI↓ lean mass↓ BP: DBP↓	^103^
	Overweight or obese adults (*n* = 20)	1 year	Body composition: body weight↓ fat mass↓ Plasma lipids: TG↓ Glucoregulatory factors: FPG↓	^129^
	Abdominally obese participants (WHtR ≥0.5) (*n* = 40)	3 months	Body composition: body weight↓ WC↓ BMI↓ WHtR↓ Glucoregulatory factors: HbA_1c_↓	^106^
	Overweight or obese female adults (*n* = 63)	12 weeks	Body composition: body weight↓ BMI↓ body fat↓ VAT mass↓ BP: DBP↓ Glucoregulatory factors: FPG↓ HOMA‐IR↓	^100^
	Overweight or obese adults (*n* = 139)	1 year	Body composition: body weight↓ fat mass↓ BP: SBP↓ DBP↓ Plasma lipids: TC↓ LDL‐c↓ TG↓ HDL‐c↑ Glucoregulatory factors: FPG↓ HOMA‐IR↓	^107^
10 h TRF	Overweight adults with T2DM (*n* = 120)	12 weeks	Body composition: body weight↓ Plasma lipids: TC↓ LDL‐c↓ TG↓ Glucoregulatory factors: HbA_1c_↓	^114^
eTRF (06:00–15:00)	Healthy participants (*n* = 82)	5 weeks	Body composition: body weight↓ Glucoregulatory factors: FPG↓ HOMA‐IR↓ Adipose factor: ghrelin↑ Inflammatory biomarkers: IL‐8↓ TNF‐α↓	^109^
eTRF (8:00–16:00)	Healthy male participants (*n* = 16)	2 weeks	Body composition: body weight↓ Glucoregulatory factors: insulin sensitivity↑ insulin↑ The Matsuda insulin sensitivity index↑	^113^
eTRF (7:00–15:00) + ER	Overweight or obese adults (*n* = 90)	14 weeks	Body composition: body weight↓ BP: DBP↓	^108^
5:2 diet	Overweight or obese adults (*n* = 112)	1 year	Body composition: body weight↓ Plasma lipids: TC↓ HDL‐c↓ Glucoregulatory factors: HbA_1c_↓	^119^
	Overweight or obese adults (*n* = 150)	50 weeks	Body composition: body weight↓	^120^
	Overweight or obese adults (*n* = 146)	1 year	Body composition: body weight↓	^121^
	Overweight or obese adults (*n* = 300)	6 months	Body composition: body weight↓	^122^
	Overweight or obese participants with hypertension (*n* = 205)	6 months	Body composition: body weight↓ fat mass↓ BP: SBP↓ DBP↓	^124^
	Obese male war veterans (*n* = 24)	6 months	Body composition: body weight↓ BMI↓ BP: SBP↓	^125^
	Participant with T2DM (*n* = 137)	1 year	Body composition: body weight↓ BMI↓ Plasma lipids: TC↓ LDL‐c↓ TG↓ HDL‐c↓ Glucoregulatory factors: HbA_1c_↓	^118^
	Participant with MetS (*n* = 39)	8 weeks	Body composition: body weight↓ BMI↓ VAT index↓ Glucoregulatory factors: fasting insulin↓ HOMA‐IR↓ Adipose factor: leptin↓ adiponectin↑ Oxidative stress: MDA↓	^123^
Ramadan fasting	Healthy male participants (*n* = 160)	1 month	Body composition: body weight↓ BMI↓ WHtR↓ fat mass↓ Plasma lipids: TG↓ Glucoregulatory factors: FPG↓	^126^
	Obese male adults (*n* = 30)	1 month	Body composition: body weight↓ BMI↓ WHtR↓ fat mass↓ Adipose factor: leptin↓	^127^
	Obese male adults (*n* = 28)	1 month	Body composition: body weight↓ BMI↓ WHtR↓ fat mass↓ Inflammatory biomarkers: IL‐6↓ TNF‐α↓	^128^

Abbreviations: ADF, alternate day fasting; ALMI, appendicular lean mass index; BMI, body mass index; BP, blood pressure; DBP, diastolic blood pressure; ER energy restriction; eTRF, early TRF; FPG, fasting plasma glucose; HbA_1c_, hemoglobin A1c; HDL‐c, high‐density lipoprotein cholesterol; HOMA‐IR, homoeostasis model assessment‐estimated‐insulin resistance; hs‐CRP, high sensitivity C‐reactive protein; IL, interleukin; LDL‐c, low‐density lipoprotein cholesterol; MDA, malondialdehyde; MetS, metabolic syndrome; SBP, systolic blood pressure; TC, total cholesterol; TG, triglyceride; TNF, tumor necrosis factor; TRF, time‐restricted feeding; T2DM, type 2 diabetes mellitus; VAT, visceral fat; WC, waist circumference; WHtR, waist–hip ratio; ↓, decrease in the indicated parameter; ↑, increase in the indicated parameter.

##### Long‐term or prolonged fasting

Fasting for 2–21 days or more.^44^ As early as the 1970s, the zero‐calorie diet (a form of LF) was used to treat patients with extreme obesity.^130^ Subsequently, water fasting and Buchinger fasting replaced the zero‐calorie diet, which had considerable side effects and demonstrated utility in the treatment of chronic diseases, such as T2DM,^131^ NAFLD,^132^ MetS,^133^ and CVD.^134^ For example, a study on 1610 patients with hypertension demonstrated that a 4–41‐day Buchinger fast markedly lowered BP, with an increased duration leading to a greater decrease in BP.^135^ Another large study involving 1422 subjects demonstrated considerable improvements in cardiometabolic parameters, including weight and BP, lipid, and blood glucose levels, as well as a considerable increase in mood stability and well‐being in subjects who received the LF regimen.^136^ Overall, medically supervised LF is an effective and safe form of fasting for treating CMD.^137^ However, it is strongly recommended that LF or similar fasting interventions be performed only under the supervision of a medical professional.

##### Fasting‐mimicking diets

A low‐calorie low‐protein diet for 5 consecutive days per month, recommended for 1–6 months per year.^45^ FMD was developed due to a series of studies on the effects of periodic LF in animal models. Studies on rodents have demonstrated that FMD prolongs the lifespan, reduces inflammation, inhibits immune senescence, modulates gut microbiota, and promotes neural regeneration and cardiac injury repair.^45^, ^138^, ^139^ In humans, a pilot clinical trial showed that three monthly FMD cycles reduced risk factors/biomarkers related to aging, diabetes, CVD, and cancer, including body weight, serum glucose, insulin‐like growth factor‐1 (IGF‐1), trunk fat, and others, without major adverse effects.^140^ Sadeghian et al.^141^ found that FMD was more effective at reducing insulin resistance and regulating appetite‐regulating hormones as well as preserving muscle mass and BMR among metabolically healthy obese women. Recently, a proof‐of‐concept study revealed that FMD can also improve HOMA‐IR and soluble urokinase plasminogen activator receptor in patients with T2DM and diabetic nephropathy, effectively inhibiting the development of T2DM and its complications.^142^ This periodic dietary strategy offers comparable benefits to CR while effectively avoiding the risk of malnutrition, thereby possessing great potential in promoting cardiometabolic health.

#### 2.1.3 Dietary protein restriction

The ratio of dietary nutrients also influences metabolic health. Early and recent studies on nutrient‐specific restrictions in animal models have demonstrated that reducing the intake of dietary protein optimized and extended the lifespan, independent of calorie intake.^143^, ^144^, ^145^ An increasing number of studies have demonstrated a direct link between PR and CMD and have suggested a beneficial effect of low‐protein diets on obesity, T2DM, and MetS^46^ A PR diet is often defined as a dietary pattern that reduces the protein intake in the diet without changing the calorie intake.^46^ Mice fed the PR diet exhibited better body weight and fasting blood glucose, insulin, and HOMA‐IR values than mice fed other diets. In humans, the PR diet considerably improved physical parameters, blood glucose and lipid levels, energy expenditure, and insulin sensitivity in patients suffering from obesity and MetS.^146^ Additionally, studies on patients with obesity have demonstrated that a high protein intake during weight loss impairs insulin signaling in muscles and normal glucose uptake rates.^147^ Maintaining a low protein intake can ensure efficient weight loss outcomes and glycemic management among patients with obesity.

#### 2.1.4 Dietary carbohydrate restriction

Growing research shows that excessive carbohydrates in the diet lead to endocrine dysregulation marked by hyperinsulinemia, promote the deposition of calories in fat cells, and thereby induce CMDs, such as obesity and T2DM, by increasing hunger and slowing metabolic rate.^148^, ^149^ Therefore, restriction of carbohydrate intake is important for improving cardiometabolic health. Low‐carbohydrate diet (LCD) is the predominant form of carbohydrate restriction, and is defined as a diet that has a low proportion of daily calories (<26%) derived from carbohydrates or contains <130 g of carbohydrate per day.^47^ LCD has long been considered an important treatment option for diabetic patients, significantly reducing postprandial blood glucose spikes and suppressing insulin secretion.^150^ As research progresses, LCDs also show more potential metabolic benefits, including reduced body fat mass, improved pre‐meal insulin sensitivity, and optimized lipid profiles. A recent meta‐analysis of the LCD in T2DM patients with >1350 participants revealed that when compared to control diets at 6 months, the LCD produced greater rates of T2DM remission, and showed improvements in weight loss, fasting insulin sensitivity, HbA_1c_, and TG. Also, with appropriate pharmacological interventions (insulin), LCD can achieve better weight control and lipid levels.^151^ In addition, researchers found that LCD significantly improved the lipid profile of patients with T2DM, lowering TG‐rich lipoproteins and LDL_5_ and increasing HDL_2_/HDL_3_, while effectively reducing intrahepatic lipid deposition.^152^ Other studies have also shown that short‐term LCDs are more adherent than very low‐carbohydrate and high‐carbohydrate diets and are more effective in improving lipid profile disorders and reducing daily blood glucose fluctuations.^151^, ^153^, ^154^ In addition, it has been demonstrated that LCD enhances fat oxidation, reduces TG, BP, and blood glucose, increases HDL‐c levels, and improves LDL phenotype in obese MetS patients,^155^ and can be combined with exercise to enhance cardiopulmonary adaptability and cardiometabolic status in obese patients.^156^, ^157^ This scientific evidence supports the potential benefit of short‐term LCD in reducing cardiometabolic risk, particularly in patients with T2DM, but its long‐term benefits are not optimal, depending on the quality of carbohydrate consumed, and may raise safety concerns such as hypoglycemia, malnutrition, disturbances in the gut microbiota, ketosis, etc.^47^, ^158^ Therefore, it is mainly recommended that some obese diabetic patients follow a short‐term high‐quality LCD diet under medical supervision and optimize it appropriately according to their nutritional and physical status.

#### 2.1.5 Dietary fat restriction

Observations on high‐saturated fat and high‐cholesterol diets with CAD led to the development of a dietary regimen to restrict dietary fat intake, which led to the development of a low‐fat diet (LFD) nutritional intervention strategy.^159^ LFD refers to a dietary pattern in which dietary fat provides 20%–30% of the total daily calorie intake. It is usually achieved by offering specific menus that emphasize low‐fat foods, or patients can be asked to count fat grams rather than calories.^48^ Current evidence suggests that LFD mainly plays a positive role in weight loss and improving body composition. For example, data from the Diabetes Prevention Program show that LFD reduced the incidence of T2DM by 58% in overweight/obese patients with abnormal glucose tolerance compared to metformin treatment, and resulted in a weight loss of 5.6 kg over an average treatment period of 2.8 years.^160^ Results of several meta‐analyses have also shown that restricting total fat intake may significantly improve body fat and lipid levels in overweight/obese patients, including reductions in body weight, BMI, WC, body fat percentage, TC, and LDL‐c levels. In particular, restriction of saturated fat is effective in reducing cardiovascular events.^161^, ^162^, ^163^ However, in other reports, compared with other diets such as LCD and Mediterranean diet, the metabolic benefits of LFD are not significant, and the long‐term effects of weight loss were inconsistent.^164^, ^165^, ^166^, ^167^, ^168^ Thus, it is generally not the first choice for patients with CMD. Like PR and LCD, LFD is a dietary pattern that improves metabolism by adjusting the proportion of macronutrients in the diet. Our diets contain a complex range of macronutrients, therefore reducing the proportion of one macronutrient alone (e.g., dietary fat), the daily caloric contribution of the other macronutrients (such as carbohydrates and protein) must be increased accordingly. This can lead to an opposite trend in overall calorie intake, making it difficult to achieve the ideal therapeutic effect. Therefore, when implementing PR, LCD, and LFD, extra attention should be given to the overall calorie intake.

It is widely known that eating less and moving more are good for our health. However, when this mantra is translated into a dietary strategy, it involves more than just fasting. Results from studies on animal models to studies on humans provide strong evidence that dietary restriction regimens improve cardiometabolic health and offer a variety of options for dietary management in patients with CMD. However, the small sample size and short intervention duration of some studies make statistical analyses of relevant results somewhat limiting. In dietary restrictions, CR focused more on improvements in body composition and weight, and therefore had a more positive effect on obesity‐related cardiovascular outcomes. IF demonstrated beneficial effects on several metabolic factors, with a good health effect in patients with obesity, T2DM, and high CMD risk. We also discussed some dietary patterns that restrict macronutrients, such as PR, LCD, and LFD, but there is significant heterogeneity in the current evidence, with benefits depending on the quality and food source of macronutrients,^169^ and more research is still needed to determine their impact on improving cardiometabolic health. It is worth noting that strict dietary restrictions such as CR and IF may increase the risk of hypoglycemia and malnutrition events in elderly patients, patients with low BMI and T2DM patients on insulin and potent hypoglycemic drugs.^170^, ^171^ Therefore, dietary restriction strategies need to be adapted to the patient's health status and medication regimen in a comprehensive manner.

### 2.2 Traditional regional diet

The results of several epidemiological surveys, prospective cohort studies, and large RCTs have shown that populations in many regions such as the Mediterranean coast, Northern Europe, Japan, and Southern China, generally have a lower prevalence of CMD and a higher lifespan,^172^, ^173^, ^174^, ^175^, ^176^ which may be related to their healthy dietary patterns based on local culture, customs, and food resources. These healthy diets have a very similar dietary structure. Hence, we have described in detail the dietary patterns of these regions to fully understand their cardiometabolic potential and to try to explore the possibility of developing dietary patterns for different regional populations that better match local dietary habits. Special food compositions included in traditional regional diets are summarized in Table 4.

**TABLE 4:** Summary of the characteristics included in the Mediterranean, Nordic, Japanese, dietary approaches to stop hypertension (DASH), Mediterranean‐DASH intervention for neurodegenerative delay (MIND) diets

Food consumption		Mediterranean diet^177^	Nordic diet^178^	Japanese diet^179^	DASH diet^180^	MIND diet^181^
Encourage food	Whole grain	1–2 servings/meal	For every meal (bread: 4–6 slices/day, cereal: 1.5 servings/day, β‐glucan‐rich foods: 3 g/day, whole grain pasta: 3 servings/week)	Rice: ≥3 bowls/day	7–8 servings/day	≥3 servings/day
	Fruits	1–2 servings/meal	Fruit, berries (blueberry and lingonberry)	≥1.8 servings/day	4–5 servings/day	Berries: ≥2 servings/week
	Vegetables	≥2 servings/meal	Vegetables, root vegetables: ≥500 g/day	≥5.4 servings/day; mushroom: ≥5 times/week	4–5 servings/day	Green leafy: ≥6 servings/week; other vegetables: ≥1 servings/day
	Oils and fat	Olive oils for every meal	Rapeseed oil: 0.5 dl/day	–	2–3 servings/day (soft margarine/mayonnaise/light salad dressing)	Olive oil for every meal; butter, margarine: <1 T/day
	Nuts and legumes	Nuts: 1–2 servings/day; legumes: ≥2 servings/week (use in combination)	Mainly almonds: 15 g/day	Soy products: ≥6 times/week	4–5 servings/week	Nuts: ≥5 servings/week; legumes: >3 servings/week
	Dairy products and cheese	1–2 servings/day (low fat)	≤5 dl/day (low‐fat milk); cheese: <17% (for cooking)	–	2–3 servings/week (low fat or skim)	Cheese: <1 servings/week
Moderate intake food	Fish and sea food	≥2 servings/week	3–5 servings/week	≥3 times/week	Meat, poultry, and fish: ≤2 servings/day	≥1 servings/week
	Eggs and white meat (poultry, turkey, rabbit, etc.)	QqqEggs: 2–4 servings/week; white meat: 2 servings/week	Eggs: as long as the intake of cholesterol did not exceed the recommended intake(RI); meat: ≤500 g/week; poultry: ≤300 g/week	–	–	Poultry: ≥2 servings/week
	Wine	Female: 1 glass/day; male: 2 glass/day	Subjects habitual amount	–	–	1 glass/day
Avoid food	Red meats and processing meats	Red meats: <2 servings/week; processing meats: <1 serving/week	–	<4 times/week	<2 servings/week (lean meat)	<4 servings/week
	Candies, pastries, and beverages	Less as much as possible	For weekends	Japanese confectionery: ≥2 times/week	Sweets: ≤5 servings/week	<5 servings/week
Special attention		–	Juice from fruits, berries, or vegetables: 4 dl/week; low alcohol beer: one bottle (33 cl/day)	Green tea: ≥2 cups/day; miso‐soup: ≥2 bowls/day; pickles: ≥6 times/week	Sodium: ≤2400 mg/day	–

#### 2.2.1 Mediterranean diet

The Mediterranean diet, which began in the early 1960s as a popular diet among people living in the Mediterranean basin, has evolved into a modern diet characterized by a high intake of virgin olive oil, whole grains, nuts, fruits, vegetables, and legumes, a moderate intake of fish, seafood, dairy products, and red wine, and a reduced consumption of red meat, processed meat, and sugar.^177^ The Mediterranean diet is known for its anti‐CVD effects, which were first reported by Keys et al.^13^ Subsequent data from several large cohort studies and RCTs have provided additional and stronger evidence of the health effects of the Mediterranean diet. They have demonstrated improvements in several risk factors and diseases,^182^, ^183^, ^184^, ^185^ as shown in Table S1. For example, the landmark PREDIMED study demonstrated an approximately 30% reduction in the risk of myocardial infarction, stroke, cardiovascular death, and new‐onset T2DM in high cardiovascular risk patients who received a 4.8‐year Mediterranean diet intervention.^186^, ^187^ The study demonstrated improved lipoprotein function and increased anti‐inflammatory and antioxidant capacities.^188^, ^189^, ^190^, ^191^ The recently published CORDIOPREV study also demonstrated that a 7‐year Mediterranean diet intervention reduced the incidence of cardiovascular events by 33% in patients with CVD.^167^ These patients also showed significant improvements in endothelial dysfunction and endothelial homeostasis and a significant reduction in the risk of atherosclerosis.^192^, ^193^ These favorable effects on known risk factors may partially explain the benefits of the Mediterranean diet on the morbidity, recurrence, and mortality of CVD. Additionally, data from DIRECT‐PLUS suggest that a green Mediterranean diet rich in polyphenols and plant proteins exhibited benefits beyond those of a traditional Mediterranean diet. These benefits include a reduction in central obesity and liver fat, modulation of the gut microbiome, reduced insulin resistance, and reduced incidence of lipid metabolism disorders.^194^, ^195^, ^196^ By adjusting the proportions and types of foods in the traditional Mediterranean diet, new dietary patterns have also been derived that produce cardiometabolic benefits comparable to or even better than those of the Mediterranean diet. For example, the Indo‐Mediterranean diet contains more whole grains, including millets, porridge, and green beans, increases a variety of healthy spices such as turmeric, cardamom, cinnamon, cumin, black pepper, cloves, and reduces the amount of animal foods.^197^ In Indo‐Mediterranean Diet Heart Study, the Indo‐Mediterranean diet effectively improved cardiometabolic risk factors such as BMI, BP, fasting glucose, and lipid profile in high‐risk patients with CAD and reduced the total cardiac end points such as myocardial infarction and sudden cardiac death, achieving more effective primary and secondary prevention of CAD.^198^ Results of a meta‐analysis also showed that treatment with Indo‐Mediterranean diet was linked to a significant decrease in all‐cause mortality and CVDs, such as heart failure and arrhythmias.^199^ It is proposed that the rich antioxidants in the Indo‐Mediterranean diet may explain its better anti‐inflammatory and cardioprotective effects.^200^

Unlike dietary restrictions, the Mediterranean diet offers a healthy dietary paradigm, with benefits dependent on patient adherence and food choices. Current evidence suggests that a higher adherence to the Mediterranean diet is associated with lower CVD risk, lower T2DM risk, and healthier cardiometabolic indices.^174^, ^201^, ^202^, ^203^ Adherence to this plant‐based dietary pattern may significantly decrease the risk of symptoms and death in patients with CMD, especially in those with CVD.

#### 2.2.2 Nordic diet

The Nordic diet is a dietary pattern that combines the Nordic nutrition recommendations, which are issued by five Nordic countries (Denmark, Finland, Iceland, Norway, and Sweden) with traditional Nordic foods. It emphasizes traditional, environmentally sustainable, and locally sourced healthy foods that encourage a high intake of leafy and root vegetables, berries, whole grains, fatty fish, legumes, and canola oil.^204^ Increasing clinical evidence suggests that the cardiometabolic health benefits of the Nordic diet and its various iterations (e.g., Healthy Nordic Diet, New Nordic Diet) are at least equivalent to those of the Mediterranean diet.^205^

The Swedish NORDIET study demonstrated, for the first time, that the Nordic diet reduces weight and decreases TC, LDL‐c, BP, and HOMA‐IR levels in patients with mild hypercholesterolemia.^178^ Subsequent trials, such as SYSDIET and Sysdimet from other Nordic regions, have concluded the same. Adherence to a healthy Nordic diet improves lipid profiles, BP, and inflammation^206^, ^207^, ^208^ and is associated with lower risks of CVD and T2DM.^209^, ^210^ In addition, the OPUS study revealed that a 6‐month New Nordic Diet significantly improved weight and BP in centrally obese patients,^211^ with high adherence and low weight regain in the subsequent 12 months of follow‐up.^212^ Analysis of blood plasma metabolomics further confirms that the long‐term metabolic benefits of New Nordic Diet may be related to its promotion of higher levels of vaccenic acid and 3‐hydroxybutanoic acid production in the body.^213^ A recent secondary analysis of the SYSDIET study demonstrated that the Nordic diet group maintained lower TC and saturated and unsaturated fat levels and exhibited better glycemic regulation, while the body weight remained largely unchanged, even after consuming more food.^214^ These findings contribute to the plausible explanations of cardiometabolic benefits, other than weight loss, induced by the Nordic diet. Further research is needed to elucidate the additional metabolic benefits of the Nordic diet and their underlying mechanisms.

#### 2.2.3 Traditional Asian diets

For quite some time, studying European diets, especially the Mediterranean diet, has been the focus. However, regional dietary patterns from other parts of the world that follow similar principles have also demonstrated positive health outcomes and deserve our attention.

The traditional Jiangnan diet comes from the lower Yangtze River in China. It is characterized by large portions of seasonal fruits and vegetables, freshwater fish and shrimp, soybean products, moderate amounts of unrefined carbohydrates, such as rapeseed oil and brown rice, and a light and oily cooking method.^215^ Studies have demonstrated that the benefits of the traditional Jiangnan diet for reducing weight, BP, and blood glucose levels are comparable to those of the Mediterranean diet and that the benefits for preventing hypoglycemia and maintaining nocturnal glucose homeostasis are superior to those of the Mediterranean diet.^216^ Other studies have demonstrated that the Jiangnan diet is also effective in reducing loss of muscle mass, preventing sarcopenia, and promoting healthy aging compared with other Chinese diets that allow the consumption of red meat and beans.^217^

The traditional Japanese diet, “washoku,” consists of one bowl of rice, one bowl of soup, and one main and two side dishes. It mainly includes rice, large amounts of vegetables, fruits, and miso soup, and a moderate amount of fish, soy products, kimchi, and seaweed.^179^, ^218^ Several studies have demonstrated the cardiometabolic potential of the Japanese diet. For example, studies have demonstrated that adherence to a Japanese diet‐based nutrition education program improved body weight, LDL‐c, oxidized LDL, and TG levels among middle‐aged men and helped convert serum phospholipid fatty acids to anti‐atherosclerotic characteristics.^219^, ^220^ In addition, the Japanese diet reduces the LDL‐c and leptin/adiponectin ratios in patients with an abnormal LDL‐c level, effectively reducing the risk of adipose tissue inflammation and atherosclerosis.^221^ Data from several other cohort studies suggest that the traditional Japanese diet may provide adherence‐related cardiometabolic benefits. The higher the adherence to the traditional Japanese diet, the lower the cardiovascular risk factors, such as BP and lipid levels,^222^, ^223^, ^224^ and the risk of death from CVD, stroke, and ischemic heart disease.^225^, ^226^ However, it has also been shown that long‐term adherence to the Japanese diet can cause excessive sodium intake and hypertension.^227^ Therefore, it is important to focus on the intake of foods high in sodium, such as miso soup and natto, when implementing the traditional Japanese diet.

A regional diet incorporates the agriculture, food industry, economy, and culture of a region. The key dietary feature that gives it cardiometabolic potential is not any particular regional cuisine but a flexible and healthy dietary structure, including rich plant foods, whole grains, nuts, moderate amounts of dairy products, fish, and small amounts of refined processed foods and red meat. The specific food choices can be integrated and adapted to the local context, consistent with the characteristics of the dietary pattern.

### 2.3 Diet based on the control of macronutrient content or foods

In addition to dietary restrictions and regional diets, there is another type of dietary pattern that derives from the additional emphasis on nutrients and foods that constitute. For example, the plant‐based diets (PBDs) with an emphasis on plant products, DASH diets with an emphasis on low salt and sodium, KD with an emphasis on ketone production, and Mediterranean‐DASH intervention for neurodegenerative delay (MIND) diets with an emphasis on food/s that improve cognitive components. Here, we summarize these dietary patterns, describing their impact on cardiometabolic health and thus providing additional nutritional treatment options for people with CMD.

#### 2.3.1 Plant‐based diets

PBDs are a diverse group consisting of vegan, lacto‐ovo‐vegetarian, and semi‐vegetarian diets. It is characterized by the maximum intake of plant products and reducing or eliminating animal‐based food consumption.^228^ Data from large prospective cohort studies, such as EPIC‐Oxford, TCHS, AHS‐2, and IMS, have consistently demonstrated that vegetarians exhibit lower all‐cause mortality, cardiovascular‐related mortality, and less cardiometabolic risk than meat eaters.^229^, ^230^, ^231^ This may be attributed to the beneficial effects that PBD has on multiple cardiometabolic risk factors. Several RCTs have demonstrated that vegetarian diets reduce body weight, fat mass, blood glucose, and lipid and inflammatory marker levels and improve pancreatic β‐cell function in patients with CMD.^232^, ^233^, ^234^ For example, the BROAD study shows that adhering to a vegetarian diet led to significant improvements in BMI, body weight, LDL‐c, and HbA_1c_ in obese patients, reduced pharmacological interventions and improved overall quality of life.^235^ Another trial demonstrated a significantly greater reduction in hs‐CRP with a vegan diet in patients with established CAD on guideline‐directed medical therapy.^236^ A plant‐based vegetarian diet may be an adjunctive treatment to reduce the risk of reoccurrence of CAD. It has also been demonstrated that some dietary components of PBD inhibit oxidative stress injury, endothelial dysfunction, and gut microbiota disorders.^237^ These positive results from RCTs, in association with the low CMD risk found in prospective cohort studies, provide strong evidence for the cardiometabolic benefits of PBD, as shown in Table S2. However, there is considerable heterogeneity in the cardiometabolic effects of different PBD qualities and subtypes. High‐quality PBDs are independently and negatively associated with the development of obesity, CVD, and T2DM. In contrast, low‐quality PBDs (e.g., diets rich in refined grains and French fries) were associated with an increased risk of CVD.^238^ The focus of a cardiometabolically beneficial PBD is not only on limiting animal foods but also on improving the quality of its plant‐based components.

#### 2.3.2 Ketogenic diet

A KD is a formula diet that is high in fat and very low in carbohydrates, with moderate intake levels of protein and other nutrients. The core goal of the diet is to change how the body provides energy through strict carbohydrate restrictions, which triggers a state of nutritional ketosis.^239^ In recent years, the KD has demonstrated great promise in improving cardiometabolism.^240^ In particular, among obese individuals and patients with T2DM, KD can significantly improve weight loss, body fat mass, BMI, BP, blood glucose level, and HbA_1c_ level.^241^, ^242^, ^243^, ^244^, ^245^ It may also have additional benefits, such as the prevention of muscle loss, appetite control, and hormonal regulation.^246^, ^247^ However, KD does have some potential risks, such as an elevated LDL‐c^248^ and, in some cases, ketoacidosis and kidney disease. KD therapy for patients with CMD should be implemented after performing a comprehensive evaluation under the supervision of a medical professional.

#### 2.3.3 DASH and MIND diets

The DASH and MIND diets are dietary patterns established for the treatment of specific diseases (hypertension, cognitive impairment) and are closely related to the Mediterranean diet, with a similar dietary structure, as shown in Table 4. The DASH diet was derived from the Dietary Approaches to Stop Hypertension study, which evaluated the effects of dietary patterns on BP.^249^ Its richness in fruits and vegetables, low‐fat milk, and whole grains, moderate amounts of nuts and legumes, and reduced amounts of red meat, fats, refined sugars, and sugary drinks exhibited considerable BP‐lowering effects compared with the daily American diet.^180^ Subsequent clinical studies have further confirmed the antihypertensive effects of the DASH diet and expanded its list of positive effects, including improvements in other cardiovascular risk factors and comorbidities.^250^, ^251^, ^252^ Systematic reviews and meta‐analyses from multiple RCTs and prospective studies have demonstrated that the DASH diet considerably reduces body weight and improves the lipid profile, blood glucose level, insulin resistance, inflammatory response, and oxidative stress markers. The diet was also highly associated with lower incidence rates of CVD, stroke, heart failure, and T2DM.^253^, ^254^, ^255^ Adherence to the DASH diet considerably reduces the risk of all‐cause mortality, CVD, stroke, and cancer.^256^

Another dietary pattern, the MIND diet, combines the beneficial elements of the DASH and Mediterranean diets with a special emphasis on neuroprotective and cognitive‐improving dietary components, such as leafy green vegetables and berries.^181^ Considering that the MIND diet is composed of two diets associated with a reduced risk of CVD, it is also considered to have some potential for improving cardiometabolism. The results of two cross‐sectional studies suggest that MIND diet scores were negatively associated with the probability of lower HDL and general obesity in adults and not with abdominal obesity.^257^, ^258^ Another cohort study assessed the relationship between MIND diet adherence and CVD risk in adults. A higher MIND diet adherence was consistently associated with a lower risk of cardiovascular events, and each 1‐point increase in MIND diet score was associated with a 16% reduction in CVD incidence.^259^

## 3 POTENTIAL MECHANISM MEDIATING THE EFFECTS OF DIETARY PATTERNS

**FIGURE 3:**
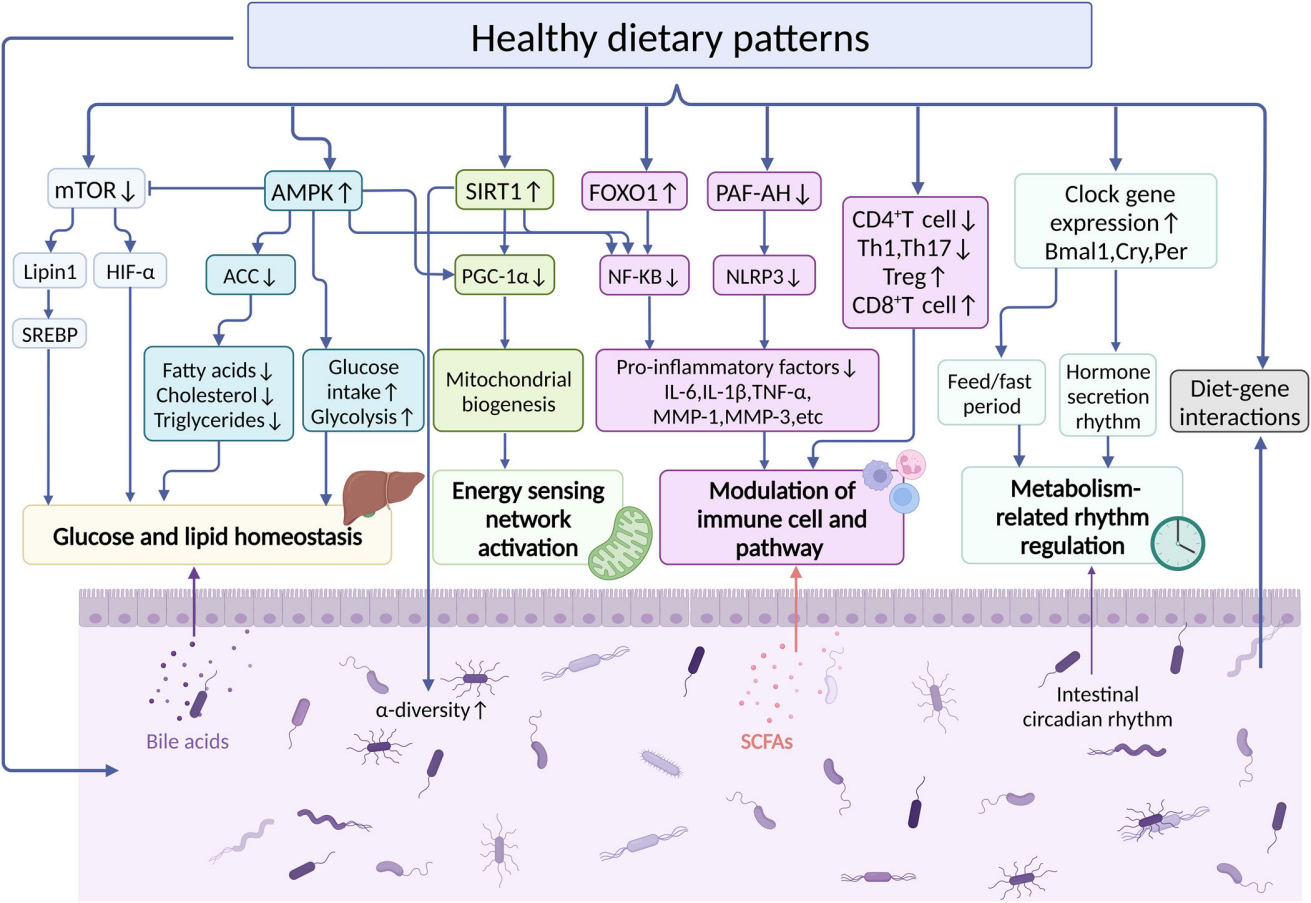
Key molecular mechanisms by which dietary patterns affect cardiac metabolism. The dietary patterns that regulate nutrient‐sensing pathways (including mammalian target of rapamycin [mTOR], AMP‐activated protein kinase [AMPK], and Sirtuin‐1 [SIRT1]), the immune system, the gut microbiome, and circadian rhythms and their associated signaling events are shown. Elucidating the mechanisms of dietary intervention on cellular stress response and host metabolic dysfunction at the molecular, cellular, and metabolite levels will help to create more precise and dynamic dietary strategies. Abbreviation: ACC, acetyl‐CoA carboxylase; FOXO1, forkhead box protein O1; HIF‐1α, hypoxia‐inducible factor‐1α; IL, interleukin; MMP, matrix metalloprotease; NF‐κB, nuclear factor‐kappa B; NLRP3, NACHT, LRR, and PYD domains‐containing protein 3; PAF‐AH, platelet‐activating factor acetylE:hydrolase; SCFA, short‐chain fatty acids; Th, T‐helper; TNF, tumor necrosis factor; Treg, regulatory T cells

The above systematic review of clinical evidence demonstrates the metabolic benefits of dietary patterns on CMD such as obesity, T2DM, and CVD. These benefits are achieved by regulating several key interrelated pathways, including regulation of nutrient‐sensing pathways to maintain glucolipid and energy balance, modulation of immune system homeostasis to suppress inflammatory responses, and improvement of the composition of the gut microbiome and restoration of disturbed circadian to promote a healthy metabolic phenotype, as shown in Figure 3. Through alterable consumption of multiple nutrients, including carbohydrates, fats, amino acids, and micronutrients. Dietary patterns activate intracellular nutritional signals and their downstream biochemical pathways, and alter the metabolic status of tissues and organs through diet–endocrine axis, diet–immune axis, diet–gut axis, and diet–nerve axis, which ultimately effectively inhibit the progression of CMD and maintain the health of the host.

### 3.1 Nutrient response pathways

#### 3.1.1 Mammalian target of rapamycin

Mammalian target of rapamycin (mTOR) belongs to the phosphatidylinositol kinase‐related kinase family. It is a serine/threonine protein kinase with a molecular weight of 289 kDa. Numerous studies have demonstrated that genetic modification, rapamycin, and dietary restrictions can inhibit mTOR overactivation, which could improve lipid and glucose homeostasis, reduce metabolic damage and aging, and prolong lifespan.^260^, ^261^ For example, studies on nutrition and aging in short‐lived organisms (e.g., yeast and worms) have demonstrated that CR can extend the lifespan by inhibiting the mTOR pathway.^262^, ^263^ Inhibitors of mTOR, such as rapamycin, have also been used as CR mimics to combat damage from aging. Another study on high‐fat diet (HFD)‐fed mice demonstrated that TRF improved the mTOR pathway function without reducing calorie intake, maintained glucose homeostasis and anabolism in the liver, and partially reversed the metabolic disorders caused by HFD.^93^ In addition, Wu et al.^264^ demonstrated that the PR diet could treat metabolic disorders by inhibiting the mTOR pathway and reducing the hunger and appetite caused by food restriction, which helps maintain the effects of dieting and weight loss.

#### 3.1.2 AMP‐activated protein kinase

AMP‐activated protein kinase (AMPK), an important kinase in regulating energy homeostasis, is one of the key regulators of energy sensing and metabolic homeostasis in eukaryotic cells and is involved in various signaling pathways, including mTOR signaling. Studies on rodents have demonstrated that excessive calorie intake downregulates AMPK activation, leading to metabolic dysregulation, inflammation, and insulin resistance.^265^ Dietary restrictions, such as CR or fasting, can regulate energy metabolism by activating the AMPK pathway, which drives lipid droplet fusion and lipolysis, thus effectively reducing the risks of obesity and related metabolic disorders.^266^, ^267^ In addition, a healthy dietary pattern rich in nutrients, including flavonoids, lycopene, and resveratrol, has also been effective in activating AMPK and its downstream pathways to improve hepatic lipid metabolism, reduce insulin resistance, and decrease inflammation and oxidative stress damage.^268^, ^269^, ^270^, ^271^

#### 3.1.3 Sirtuin‐1

Sirtuins are a class of NAD^+^‐dependent deacetylases conserved from bacteria to humans. Among them, Sirtuin‐1 (SIRT1), enriched in the nucleus, is one of the most sought‐after members and a key regulator in metabolism, immune response, and aging.^272^ In animal model studies, SIRT‐overexpressing mice exhibited phenotypes similar to those of CR mice, including a lower body weight and reduced blood lipid, glucose, and insulin levels, as well as enhanced metabolic activity and glucose homeostasis.^273^ In contrast, SIRT‐deficient mice could not adapt to the CR environment or were unresponsive to CR.^274^ This suggests that the activation of SIRT1 is closely related to the improvement in CR‐induced metabolism. In addition, fasting also induced SIRT1 activation and subsequent metabolic improvements. Lilja et al.^275^ demonstrated that periodic fasting increased SIRT1 and SIRT3 expression levels and gut microbiota diversity, reduced body weight and fat mass, and induced a ketogenic state, suggesting that fasting may regulate host metabolism by affecting gut microbiota diversity through the modulation of the SIRT1 pathway. Another study confirmed that resveratrol, which is prevalent in healthy dietary patterns, such as the Mediterranean diet, may act as a SIRT agonist to reduce the risks of obesity, T2DM, and heart failure.^276^, ^277^ In conclusion, these findings suggest that SIRT1 may play an important role in the response to dietary patterns.

Notably, the abovementioned nutrient‐sensing pathways do not act independently but are rather highly interdependent. For example, CR does not directly inhibit mTOR activity but inhibits mTOR signaling by affecting its upstream energy‐sensing complexes and by activating AMPK. SIRT‐1 interacts directly and negatively with mTOR, and the deficiency or inhibition of SIRT‐1 leads to mTOR activation. In addition, there is a crosstalk between SIRT‐1 and AMPK, and a decrease in AMPK activity effectively inhibits the response of SIRT‐1 to low energy states.

### 3.2 Immune regulation

Immune dysregulation has long been recognized as an independent risk factor for the development of CMD.^278^ As a major source of metabolic fuel, diet plays an important role in immune defense response. The body's nutritional status, dietary intake patterns, and nutritional supplements (e.g., vitamins and minerals) can positively or negatively affect immune system functions, such as innate immunity, adaptive immunity, and the microbiome.^279^, ^280^, ^281^ Some recent studies have demonstrated that CR enhances memory T‐cell function and body immunity, increases thymus volume and T‐cell output, and remodels the adipose tissue transcriptome through anti‐inflammatory, mitochondrial biosynthesis, and aging‐related pathways. These effects reduce metabolic abnormalities and prevent secondary bacterial infections.^282^, ^283^ CR‐induced hypoenergetic states also downregulate PI3K/Akt/mTOR signaling and inhibit glycolysis and T‐helper (Th)1, Th17, and M1 macrophage differentiation.^284^ Fasting also plays an important role in maintaining immune homeostasis, promoting immune memory, influencing immune cell dynamics and mucosal immune responses, and remodeling and enhancing innate immune function.^285^, ^286^, ^287^ Jordan et al.^288^ demonstrated that IF reduces the number of circulating proinflammatory monocytes in healthy humans and mice and controls blood monocyte metabolism and inflammatory activity through the activation of the AMPK/PPARα pathway. Liang et al.^289^ demonstrated that fasting therapy reduces insulin resistance in rats by inhibiting NACHT, LRR, and PYD domains‐containing protein 3 (NLRP3) inflammatory vesicles and improves glucose tolerance and fatty acid metabolism. These results strongly support the benefits of restrictive dietary patterns on the immune system. In addition, it has been demonstrated that KD affects host immunity by modulating the gut microbiota composition and suppressing adipose tissue inflammation and energy homeostasis.^290^, ^291^ Additional data from large cohort studies, such as the PREDIMED, DIRECT‐PLUS, and DASH‐Sodium trials, suggest that the Mediterranean diet, the DASH diet, and other similar diets suppress the levels of the circulating inflammatory factors hs‐CRP, interleukin (IL)‐6, and tumor necrosis factor (TNF)‐α.^195^, ^292^, ^293^ In conclusion, a healthy dietary pattern may improve CMD and related immunometabolic disorders by enhancing immunity and limiting inflammatory responses by delaying immune system aging and activating various anti‐inflammatory pathways.

### 3.3 Gut microbiota and its metabolites

The role of gut microbiota in CMD progression has been demonstrated in several model and clinical studies.^294^, ^295^ Gut microbiota and its metabolites are not only key signaling hubs in the regulation of cardiometabolism but also major risk factors for individual‐level differences in CMD prognosis. Dietary restriction or modification may counteract the metabolic damage associated with obesity and HFDs by altering the composition and function of gut microbiota.^296^ For example, CR increases the α‐diversity and species‐richness of the gut microbiome in mice, modulates the diversity and ratio of beneficial and harmful bacteria, creates a unique microbial community dominated by *Lactobacillus*, and mitigates aging‐related inflammatory damage by reducing bacterial antigen load and inflammatory response marker levels.^297^, ^298^, ^299^ In humans, CR interventions reduce the ratio of thick‐walled‐to‐bacteriophage phylum in the gut of obese adolescents, helping to restructure the microbiota to a state similar to that of lean adolescents.^300^ Additionally, CR modulates bile acid metabolism through gut microbiota, improving adipose tissue dysfunction and delaying immune aging.^301^, ^302^, ^303^ Recently, Gregor et al.^304^ compared the effects of different types of restrictive diets on the gastrointestinal tract of mice and demonstrated that IF, FMD, and KD had similar benefits to those of CR. These diets also altered the intestinal microbial composition, reducing inflammatory factor expression, improving mucus production and intestinal morphology, and regulating autophagy and mitochondrial function. IF was more advantageous in improving intestinal immunity, increasing the expression of the intestinal nuclear transcription factor‐kappa B (NF‐κB) inhibitor IKB, and decreasing circulating immune factors. In addition, several studies have demonstrated that fasting‐induced gut microbiota remodeling is also involved in regulating energy metabolism, adipose tissue browning, and the brain–gut–microbiome signaling axis.^305^, ^306^, ^307^ Liu et al.^308^ demonstrated that ADF improved cognitive performance in diabetic mice by remodeling the microbiota composition, increasing the content of microbial metabolites butyrate, acetate, and short‐chain fatty acids (SCFAs), and regulating insulin signaling and brain‐derived neurotrophic factor expression. Another study also demonstrated that fasting‐induced changes in the functional aspect of gut microbiota improved BP control and metabolism by affecting SCFA production.^309^ In addition, KD is important for enriching beneficial intestinal microbiota (e.g., *Bacteroides* phylum) to improve metabolic profiles.^310^ Ang et al.^290^ demonstrated that 4 weeks of KD affected the intestinal proinflammatory Th17 cell levels by altering the gut microbial structure—decreasing the abundance of *Actinobacteria* and thick‐walled *Bacteroides* and increasing the abundance of *Bacteroides*—in patients with obesity. The alteration ultimately causes changes in the intestinal immune environment and even in the body's immune response. Another large retrospective study confirmed that increased adherence to the Mediterranean diet and PBD mitigates the loss of gut microbiome diversity, reduces frailty, metabolic disorders, and inflammatory aging,^311^, ^312^ and suppresses gut‐dependent metabolites associated with high cardiometabolic risk, such as trimethylamine‐N‐oxide (TMAO).^313^, ^314^

**FIGURE 4:**
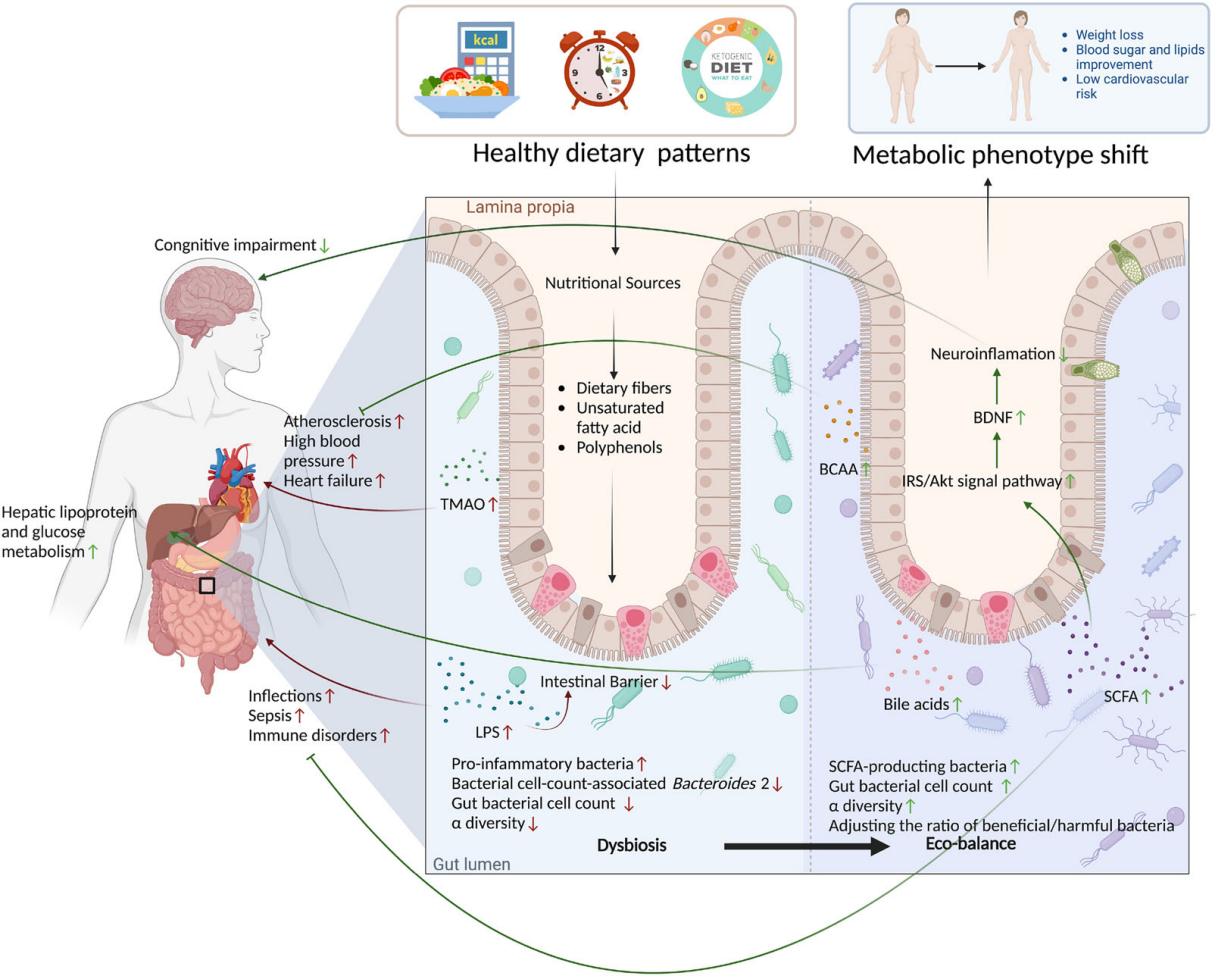
Schematic diagram of diet–gut microbiota–host metabolism. This figure depicts the direct effects of healthy dietary patterns on the gut microbiota to influence host metabolic phenotypes. This includes increasing gut microbiota diversity, adjusting the ratio of beneficial to harmful bacteria, and promoting increased secretion of the beneficial microbial metabolites short‐chain fatty acids (SCFAs) and branched‐chain amino acids (BCAAs). Abbreviation: BDNF, brain‐derived neurotrophic factor; LPS, lipopolysaccharide; TMAO, trimethylamine oxide

From the above review of recent evidence, it is easy to conclude that dietary patterns can participate in various aspects of metabolic regulation by shaping the gut microbiome, as shown in Table S3. Gut microbiota can also influence host metabolic phenotypes by inversely affecting appetite and dietary preferences.^315^, ^316^, ^317^ Thus, the benefits of dietary patterns on cardiometabolism may be far greater than we thought, even beyond genetic and environmental factors, as shown in Figure 4.

### 3.4 Circadian rhythms

Disturbed circadian rhythms are a distinctive feature of CMDs, such as obesity, T2DM, and atherosclerosis, and are closely related to poor dietary habits.^318^ There is increasing evidence that properly timed meals can independently drive rhythms of gene expression that mediate nutrient metabolism in mice with abnormal biological clocks and reverse the metabolic damage caused by rhythmic disruptions.^319^, ^320^ For example, Chaix et al.^321^ demonstrated that TRF effectively ameliorated metabolic defects in *Cry1;Cry2* (CDKO) and liver‐specific Bmal1 and Rev‐erbα/β‐knockout mice, thereby preserving circadian rhythms in liver transcripts and nutrient‐sensing pathways. Desmet et al.^322^ demonstrated that TRF selectively prevents jet lag‐induced disruption of the central biological clock in mice and regulates the normal rhythm of food intake to prevent weight gain. This suggests that proper feeding/fasting cycles can coordinate or reshape the biological clock to regulate behavioral and metabolic rhythms. In addition, the gut microbiome has a complex bidirectional regulatory role with the circadian system. The rhythmic oscillations of microorganisms are the basis for their time‐specific functions, including promoting digestion and energy metabolism during the daytime or active period, and detoxification during the nighttime or resting period.^323^ TRF has been demonstrated to affect the cyclic fluctuations of the cecum microbiota. Recently, Machado et al.^324^ demonstrated that TRF also maintains the circulation dynamics and transcript levels of the ileal microbiota, restores the ileal circadian rhythm and intestinal dynamics disturbed by HFD, and improves ileal bile acids and Farnesoid X receptor signaling. This suggests that modulation of feeding rhythms can drive circadian oscillations among microbial communities and secondary metabolites in the luminal environment of the gut, contributing to the maintenance of peripheral circadian clock entrainment and host metabolic rhythms. In addition, some nutrients affect circadian clock gene expression. For example, resveratrol, omega‐3 fatty acids, and caffeine have been demonstrated to influence host circadian clock rhythms and improve their overall metabolic status.^325^, ^326^, ^327^ Therefore, considering the appropriate timing of food intake and the diurnal distribution of dietary calories is essential to limit cardiometabolic risk.

## 4 CONCLUSION AND PROSPECTIVE

The impact of food on health has been an important topic throughout human history. In this review, we summarized cutting‐edge developments between restrictive diets, regional diets, and several dietary patterns based on controlled macronutrients and food groups and CMD, demonstrating the appeal of dietary patterns across multiple dimensions in improving cardiometabolic health. However, the lack of large case–control and long‐term longitudinal cohort studies may prevent us from determining how these dietary patterns can prevent CMD or slow its development. More in‐depth work is still needed, such as long‐term, large‐sample size, and cross‐regional prospective studies, more accurate and rapid dietary assessment questionnaires and tools, and unraveling the specific mechanisms by which dietary patterns affect CMD at the genetic, molecular, microbiota, and metabolite levels.

For nearly a century, individual response differences to dietary components have been scientifically validated by numerous diet and omics studies, such as the inclusion of genetic variants such as Pkn in nutrient‐mediated responses^328^ or how differences in gut microbial community characteristics affect glycemic response.^329^ Therefore, providing more precise and dynamic personalized nutritional advice based on gene–diet interactions than is currently available should be a priority and an important direction for future nutritional health policy development. Gut microbiota is a novel player in the pathophysiology of CMD and a predictor of individual response to dietary interventions. In the future, we can optimize nutrient ratios based on the microbiota profile of CMD patients. For example, we can recommend a high‐fiber diet for T2DM patients,^330^ measure stable microbial metabolites (e.g., TMAO) for risk stratification and the subgroup management of CMD,^331^ or create personalized dietary patterns with computer algorithms by integrating gut microbiome data.^332^, ^333^ The aim is to have precise nutritional and metabolic regulation of CMD.

We are now entering an exciting era of nutritional science. The scientific findings of the present study and the literature support the use of dietary strategies to prevent, slow, and even reverse CMD. Moreover, based on the understanding of the complex interactions between diet, gut microbiota, and CMD, as well as the development of new model systems and emerging tools in modern biology, low‐cost, personalized dietary interventions that offer precise nutritional care and treatment solutions may become available to the public.

## AUTHOR CONTRIBUTIONS

YL contributed to the topic design, manuscript revision, and the decision to submit for publication. WTW, YFL, BYL and YWL performed the literature retrieval, collation, analysis, and wrote the manuscript together. WTW and YFL were co‐first authors. ZXL and KJC revised the manuscript. All authors approved the final version of the manuscript.

## CONFLICT OF INTEREST

The authors declare they have no conflicts of interest.

## ETHICS STATEMENT

Not applicable.

## Supporting information

Supporting information

## Data Availability

Not applicable.
