# Supplementary material for: Dietary patterns and cardiometabolic health: Clinical evidence and mechanism
Source: MedComm (2020). 2023 Feb 5;4(1):e212. doi: 10.1002/mco2.212 (PMC9899878; doi:10.1002/mco2.212)
Supplement: Supplementary file 1 — Supporting information [file MCO2-4-e212-s001.docx]

**Dietary Patterns and Cardiometabolic Health: Clinical Evidence and Mechanism**

Wenting Wang^1#^, Yanfei Liu^1#^, Yiwen Li^1^, Binyu Luo^1^, Zhixiu Lin^2^, Keji Chen^1^ and Yue Liu^1^

1. National Clinical Research Centre for Chinese Medicine Cardiology, Xiyuan Hospital, China Academy of Chinese Medical Sciences, Beijing, China.
2. Faculty of medicine, The Chinese University of Hong Kong, HongKong.

Running title: Dietary Patterns and Cardiometabolic Health

***Corresponding author***:

Prof. Yue Liu

National Clinical Research Centre for Chinese Medicine Cardiology,

Xiyuan Hospital, China Academy of Chinese Medical Sciences,

Beijing 10091, China.

1. mail: liuyueheart@hotmail.com

**Supplementary Information**

**Table S1 Effect of Mediterranean diet on cardiometabolic risk factors in RCT**

| **Dietary**  **Intervention** | **Disease/target population** | **Follow-up time** | **Improvements in cardiometabolic health** | **Reference** |
| --- | --- | --- | --- | --- |
| MD | Health subjects (n=166) | 6 months (MedDairy) | Blood pressure: SBP ↓ | ^1^ |
| MD-VOO/Nuts | Subjects with high cardiovascular risk (n=210) | 1 year  (PREDIMED) | Plasma lipids: LDL particles against oxidation↑  LDL particles size↑  LDL oxidative modifications↓ | ^2^ |
| MD-VOO/Nuts | Subjects with high cardiovascular risk (n=296) | 1 year  (PREDIMED) | Plasma lipids: the HDL-C esterification index↑  HDL oxidation lag time↑  the content of triglycerides in HDL core↓  HDL oxidation index↓ | ^3^ |
| MD-VOO/Nuts | Subjects with high cardiovascular risk(n=358) | 1 year  (PREDIMED) | Plasma lipids: PAF-AH activity in HDLs↑b  α1-antitrypsin in HDLs↓b | ^4^ |
| Green-MD | Subjects with high cardiovascular risk(n=294) | 6 months  (DIRECT-PLUS) | Blood pressure: SBP ↓DBP↓  Plasma lipids: LDL-C↓ TG↓ TC/HDL-C↓ TG/HDL-C↓  LDL-C/HDL-C↓  Glucoregulatory factors: HOMA-IR↓ Fasting insulin↓  Inflammatory biomarkers: hs-CRP↓ | ^5^ |
| MD | Subjects with CHD(n=1002) | 7 year  (CORDIOPREV) | Reduction in major cardiovascular events (myocardial infarction, revascularization, ischemic stroke, documented peripheral artery disease and cardiovascular death) | ^6^ |

Abbreviations: MD, Mediterranean diet; MD-VOO, tradition Mediterranean diet+virgin olive oil; MD-Nuts, tradition Mediterranean diet+nuts; CHD, coronary heart disease; T2DM, type 2 diabetes mellitus; RCT, randomized clinical trial; SBP, systolic blood pressure; DBP, Diastolic blood pressure; TC, Total cholesterol; TG, Triglyceride; LDL-C, Low-density lipoprotein cholesterol; HDL-C, High-density lipoprotein cholesterol; HOMA-IR, Homoeostasis model assessment-estimated-Insulin resistance ; FPG, Fasting plasma glucose; hs-CRP, High sensitivity C-reactive protein;↓, decrease in the indicated parameter; ↑, increase in the indicated parameter.

**Table S2 Effect of PBD on cardiometabolic risk factors in RCT**

| **Disease/target population** | **Follow-up time** | **Improvements in cardiometabolic health** | **Reference** |
| --- | --- | --- | --- |
| Overweight and obese subjects (n=75) | 16 weeks | Body composition: Body weight↓ VAT volume↓  Plasma lipids: TC↓ LDL-C↓ HDL-C↓  Glucoregulatory factors: FPG↓ HOMA-IR↓ Fasting C-peptide↓ Fasting insulin↓ Mean glucose↓ | ^7^ |
| Overweight and obese subjects (n=244) | 16 weeks | Body composition: Body weight↓ BMI↓ Fat mass↓ VAT volume↓  Plasma lipids: TC↓ LDL-C↓ HDL-C↓  Glucoregulatory factors: FPG↓ HOMA-IR↓ Fasting insulin↓ | ^8^ |
| Overweight and obese subjects(n=168) | 16 weeks | Body composition: Body weight↓ BMI↓ Fat mass↓ VAT volume↓ | ^9^ |
| Obesity adults with a diagnosis of one of type 2 diabetes, ischaemic heart disease, or the cardiovascular risk factors(n=65) | 1 year  (BOARD) | Body composition: Body weight↓ BMI↓  Plasma lipids: TC↓ LDL-C↓ HDL-C↓  Glucoregulatory factors: HbA1c↓ | ^10^ |
| Participant with CAD(n=100) | 8 weeks  (EVADECAD) | Inflammatory biomarkers: hs-CRP↓ | ^11^ |

Abbreviations: PBD, plant-based diet; T2DM, type 2 diabetes mellitus; CAD, coronary artery disease; RCT, randomized clinical trial; BMI, body mass index; VAT, Visceral fat; ; SBP, systolic blood pressure; DBP, Diastolic blood pressure; TC, Total cholesterol; TG, Triglyceride; LDL-C, Low-density lipoprotein cholesterol; HDL-C, High-density lipoprotein cholesterol; HOMA-IR, Homoeostasis model assessment-estimated-Insulin resistance ; FPG, Fasting plasma glucose; hs-CRP, High sensitivity C-reactive protein;↓, decrease in the indicated parameter; ↑, increase in the indicated parameter.

**Table S3 Effect of dietary patterns on gut microbiota and metabolites in the human model**

| Dietary intervention | Gut microbiota alteration | Reference |
| --- | --- | --- |
| CR | Total gut microbiota↑  *Roseburia, Faecalibacterium and Clostridium XIVa*.↑  *Firmicutes-Bacteroidetes* ratio↓ | ^12^ |
| 5:2 diet | *Ruminococcus gnavus, Chitinophagaceae bacterium, Roseburia faecis, Paraburkholderia caribensis, Streptococcus ferus↑* | ^13^ |
| TRF | α-diversity↑  *Prevotellaceae, Bacteroideaceae,* *Lachnospiraceae, Ruminococcus, Feacalibacterium, Dialister*↑  *Firmicutes, Alloprevotella*↓ | ^14, 15^ |
| Ramadan diet | *Akkermansia muciniphila, Bacteroides fragilis*, *Butyricicoccus, Faecaliba,cterium, Roseburia, Allobaculum, Eubacterium, Dialister, Erysipelotrichi,* *Ruminococcaceae↑*  *Bifidobacterium, Enterobacteriaceae, Clostridium, Escherichia,* *Prevotellaceae*↓ | ^16-18^ |
| Mediterranean diet | α-diversity ↑  Clostridium XIVa, *Faecalibacterium Prausnitzii, Ruminococcus gnavus*↑  *Bacteroidetes, Prevotellaceae, Prevotella*↑  BCAA, SCFA production↑  Bile acids sensibility↑  *Firmicutes-Bacteroidetes* ratio↓  *Firmicutes, Lachnospiraceae*, *Bifidobacterium*↓ | ^19-21^ |
| Plant-based diet | α-diversity ↑  *Bifidobacterium*, *Prevotella, Lactobacillus*, *Ruminococcus*, *Roseburia,* *Eubacterium rectale*↑  *Clostridium clostridioforme, Faecalibacterium prausnitzii*↑  *Prevotella* to *Bacteroidetes* ratio↑  SCFA production↑  *Firmicutes-Bacteroidetes* ratio↓  *Proteobacterias, Bacteroides fragilis, Lachnospiraceae, C. perfringens*, *C. histolyticum,* *Gastric Streptococcus*↓  TMAO↓ | ^22^ |
| Ketogenic diet | α-diversity↓  *Bacteroidetes,* *Lactobacillus, Akkermansia muciniphila*↑  SCFA production↑  *Firmicutes,* *Desulfovibrio,* *Actinobacteria,* *Bifidobacterium*↓ | ^23^ |

Abbreviations: CR, calorie restriction; TRF, time-restricted fasting; BCAA, Branched-chain amino acid; SCFA, Short chain fatty acids; TMAO, Trimethylamine oxide; ↓, decrease in the indicated parameter; ↑, increase in the indicated parameter.

**Supplementary Reference**

1. Davis CR, Hodgson JM, Woodman R, Bryan J, Wilson C, Murphy KJ. A Mediterranean diet lowers blood pressure and improves endothelial function: results from the MedLey randomized intervention trial. *The American Journal of Clinical Nutrition*. 2017;105(6):1305-1313.

2. Hernáez Á, Castañer O, Goday A, et al. The Mediterranean Diet decreases LDL atherogenicity in high cardiovascular risk individuals: a randomized controlled trial. *Molecular Nutrition & Food Research*. 2017;61(9)

3. Hernáez Á, Castañer O, Elosua R, et al. Mediterranean Diet Improves High-Density Lipoprotein Function in High-Cardiovascular-Risk Individuals: A Randomized Controlled Trial. *Circulation*. 2017;135(7):633-643.

4. Hernáez Á, Castañer O, Tresserra-Rimbau A, et al. Mediterranean Diet and Atherothrombosis Biomarkers: A Randomized Controlled Trial. *Molecular Nutrition & Food Research*. 2020;64(20):e2000350.

5. A YM, E R, G T, et al. Effect of green-Mediterranean diet on intrahepatic fat: the DIRECT PLUS randomised controlled trial. *Gut*. 2021;70(11)

6. Delgado-Lista J, Alcala-Diaz JF, Torres-Peña JD, et al. Long-term secondary prevention of cardiovascular disease with a Mediterranean diet and a low-fat diet (CORDIOPREV): a randomised controlled trial. *Lancet (London, England)*. 2022;399(10338):1876-1885.

7. Kahleova H, Tura A, Hill M, Holubkov R, Barnard ND. A Plant-Based Dietary Intervention Improves Beta-Cell Function and Insulin Resistance in Overweight Adults: A 16-Week Randomized Clinical Trial. *Nutrients*. 2018;10(2):189.

8. Kahleova H, Petersen KF, Shulman GI, et al. Effect of a Low-Fat Vegan Diet on Body Weight, Insulin Sensitivity, Postprandial Metabolism, and Intramyocellular and Hepatocellular Lipid Levels in Overweight Adults. *JAMA Network Open*. 2020;3(11):e2025454.

9. Kahleova H, Rembert E, Alwarith J, et al. Effects of a Low-Fat Vegan Diet on Gut Microbiota in Overweight Individuals and Relationships with Body Weight, Body Composition, and Insulin Sensitivity. A Randomized Clinical Trial. *Nutrients*. 2020;12(10):2917.

10. Wright N, Wilson L, Smith M, Duncan B, McHugh P. The BROAD study: A randomised controlled trial using a whole food plant-based diet in the community for obesity, ischaemic heart disease or diabetes. *Nutrition & Diabetes*. 2017;7(3):e256.

11. Shah B, Newman JD, Woolf K, et al. Anti-Inflammatory Effects of a Vegan Diet Versus the American Heart Association-Recommended Diet in Coronary Artery Disease Trial. *Journal of the American Heart Association*. 2018;7(23):e011367.

12. Ruiz A, Cerdó T, Jáuregui R, et al. One-year calorie restriction impacts gut microbial composition but not its metabolic performance in obese adolescents. *Environmental Microbiology*. 2017;19(4):1536-1551.

13. Guo Y, Luo S, Ye Y, Yin S, Fan J, Xia M. Intermittent Fasting Improves Cardiometabolic Risk Factors and Alters Gut Microbiota in Metabolic Syndrome Patients. *The Journal of Clinical Endocrinology and Metabolism*. 2021;106(1):64-79.

14. Zeb F, Wu X, Chen L, et al. Effect of time-restricted feeding on metabolic risk and circadian rhythm associated with gut microbiome in healthy males. *The British Journal of Nutrition*. 2020;123(11):1216-1226.

15. Zeb F, Wu X, Chen L, et al. Time-restricted feeding is associated with changes in human gut microbiota related to nutrient intake. *Nutrition (Burbank, Los Angeles County, Calif)*. 2020;78:110797.

16. Ozkul C, Yalinay M, Karakan T. Structural changes in gut microbiome after Ramadan fasting: a pilot study. *Beneficial Microbes*. 2020;11(3):227-233.

17. Özkul C, Yalınay M, Karakan T. Islamic fasting leads to an increased abundance of Akkermansia muciniphila and Bacteroides fragilis group: A preliminary study on intermittent fasting. *The Turkish Journal of Gastroenterology*. 2019;30(12):1030-1035.

18. Su J, Wang Y, Zhang X, et al. Remodeling of the gut microbiome during Ramadan-associated intermittent fasting. *The American Journal of Clinical Nutrition*. 2021;113(5):1332-1342.

19. Rinott E, Meir AY, Tsaban G, et al. The effects of the Green-Mediterranean diet on cardiometabolic health are linked to gut microbiome modifications: a randomized controlled trial. *Genome Medicine*. 2022;14(1):29.

20. Ghosh TS, Rampelli S, Jeffery IB, et al. Mediterranean diet intervention alters the gut microbiome in older people reducing frailty and improving health status: the NU-AGE 1-year dietary intervention across five European countries. *Gut*. 2020;69(7):1218-1228.

21. Merra G, Noce A, Marrone G, et al. Influence of Mediterranean Diet on Human Gut Microbiota. *Nutrients*. 2020;13(1):E7.

22. Miao Z, Du W, Xiao C, et al. Gut microbiota signatures of long-term and short-term plant-based dietary pattern and cardiometabolic health: a prospective cohort study. *BMC medicine*. 2022;20(1):204.

23. Ang QY, Alexander M, Newman JC, et al. Ketogenic diets alter the gut microbiome resulting in decreased intestinal Th17 cells. *Cell*. 2020;181(6):1263-1275.e16.
